# Blood Gene Expression Profile Predicts Response to Antipsychotics

**DOI:** 10.3389/fnmol.2018.00073

**Published:** 2018-03-06

**Authors:** Jesus Sainz, Carlos Prieto, Fulgencio Ruso-Julve, Benedicto Crespo-Facorro

**Affiliations:** ^1^Spanish National Research Council (CSIC), Institute of Biomedicine and Biotechnology of Cantabria (IBBTEC), Santander, Spain; ^2^Bioinformatics Service, Nucleus, University of Salamanca (USAL), Salamanca, Spain; ^3^School of Medicine, Department of Psychiatry, University Hospital Marqués de Valdecilla, IDIVAL, University of Cantabria, Santander, Spain; ^4^Centro Investigación Biomédica en Red Salud Mental, CIBERSAM, Santander, Spain

**Keywords:** psychosis, schizophrenia, gene expression profiling, clinical response, prediction test

## Abstract

Antipsychotic drugs are one of the largest types of prescribed drugs and have large inter-individual differences in efficacy, but there is no methodology to predict their clinical effect. Here we show a four-gene blood expression profile to predict the response to antipsychotics in schizophrenia patients before treatment. We sequenced total mRNA from blood samples of antipsychotic naïve patients who, after 3 months of treatment, were in the top 40% with the best response (15 patients) and in the bottom 40% with the worst response (15 patients) according to the Brief Psychiatric Rating Scale (BPRS). We characterized the transcriptome before treatment of these 30 patients and found 130 genes with significant differential expression (*P*_adj_ value < 0.01) associated with clinical response. Then, we used Random Forests, an ensemble learning method for classification and regression, to obtain a list of predictor genes. The expression of four genes can predict the response to antipsychotic medication with a cross-validation accuracy estimation of 0.83 and an area under the curve of 0.97 using a logistic regression. We anticipate that this approach is a gateway to select the specific antipsychotic that will produce the best response to treatment for each specific patient.

## Introduction

Antipsychotic medications are the mainstay of schizophrenia treatment (Insel, 2010). They are also used for several other clinical conditions (i.e., other psychoses, bipolar disorder, delirium, depression, personality disorders, dementia and autism; Alexander et al., 2011; Carton et al., 2015; Roberts et al., 2016) and are therefore one of the most widely used and costly types of drugs having experienced a significant increase in overall prescription in recent years (Kantor et al., 2015). Unfortunately, only 55%–60% of first episode patients will have significantly reduced the severity of their psychopathology with adequate doses of antipsychotic drugs (Crespo-Facorro et al., 2007) and 30% of patients will fail to respond to two antipsychotics after adequate trials (Meltzer, 1997; Zhang and Malhotra, 2011; Pouget et al., 2014). Research to find predictors of the response to antipsychotic treatment is an old field of psychiatry; however, despite decades of research to find clinical biomarkers, there is not a useful molecular test available to predict the response to treatment (Prata et al., 2014). In a previous study (Crespo-Facorro et al., 2015), we analyzed the blood transcriptome of 22 schizophrenia patients before and after medication with atypical antipsychotics and we found that 17 genes had significantly altered expression after medication (*P* value adjusted < 0.05). With the goal of generating an expression profile that could predict the outcome of treatment, we characterized the transcriptome of a drug-naïve (before any dose of antipsychotic medication was taken) cohort of 37 first episode schizophrenia patients. Patients were then divided into two groups according to their response after 3 months of antipsychotic treatment according to their Brief Psychiatric Rating Scale (BPRS; Lukoff et al., 1986) total scores: the first group included the top 40% of patients who had the best response to treatment (highest absolute decrease of BPRS score) further referred to as “best-responders”, and the other group included the bottom 40% of patients with the worst response (lower absolute decrease of BPRS score) further referred to as “worst-responders” (Table 1). The transcriptomes at baseline of both groups were compared using the program Deseq (Anders and Huber, 2010) to define the genes with significant differential expression.

**Table 1:** Sociodemographic and clinical characteristics of study individuals.

Characteristics	Total		Best-Responders		Worst-Responders	
	(*n* = 30)		(*n* = 15)		(*n* = 15)	
	Mean	SD	Mean	SD	Mean	SD
Age at admission (years)	29.6	10.5	29.3	11.7	29.9	9.6
Age at psychosis onset (years)	28.6	10.4	28.7	11.7	28.5	9.4
Duration of untreated psychosis (months)	16.4	23.8	14.9	25.5	18.0	22.9
Duration of untreated illness (months)	12.0	17.8	7.9	10.9	16.1	22.3
BPRS at admission	74.1	15.4	83.5	8.9	64.8	15.0
BPRS at 3 months of treatment	34.6	11.8	29.7	5.2	39.5	14.4

## Materials and Methods

### Study Setting and Subjects

The cohort analyzed in this study was obtained from an ongoing epidemiological and 3-year longitudinal intervention program of first-episode psychosis (PAFIP) conducted at the outpatient clinic and the inpatient unit at the University Hospital Marques de Valdecilla (Cantabria, Spain). Conforming to international standards for research ethics, this study was approved by the Cantabria Ethics Institutional Review Board (IRB). Patients meeting inclusion criteria and their families provided written informed consent to be included in the PAFIP. The biological samples of patients included in the study were provided by the Valdecilla biobank.

All referrals to PAFIP were screened for patients who met the following criteria: (1) 15–60 years old; (2) living in the catchment area (Cantabria); (3) experiencing a first episode of psychosis; (4) having received no prior treatment with antipsychotic medication; (5) DSM-IV criteria for schizophrenia, schizophreniform disorder, schizoaffective disorder, or brief psychotic disorder. Patients were excluded for any of the following reasons: (1) meeting DSM-IV criteria for drug dependence; (2) meeting DSM-IV criteria for mental retardation; and (3) having a history of neurological disease or head injury. Only patients with a history of drug dependence (DSM-IV diagnosis; but not drug abuse) during the last 12 months were excluded of the study. The diagnoses were confirmed using the Structured Clinical Interview for DSM-IV (SCID–I) carried out by an experienced psychiatrist 6 months on from the baseline visit. Our operational definition for a “first episode of psychosis” includes individuals with a non-affective psychosis (meeting the inclusion criteria defined above) who have not previously received antipsychotic treatment regardless the duration of psychosis. The 37 individuals who gave written consent to their participation in the program, who fulfilled inclusion criteria at 6 months, and had mRNA samples at baseline and at 3 months, were included in our analyses.

After informed consent was signed, patients were included in a prospective, randomized, flexible-dose, open-label study (EudraCT number 2013-005399-16). We used a simple randomization procedure. At study intake, all patients were antipsychotic naïve and were randomized to aripiprazole (*N* = 17), risperidone (*N* = 20). Initial dose ranges were 5–10 mg/day of aripiprazole and 1–2 mg/day of risperidone. Rapid titration schedule (5-day), until optimal dose was reached, was used as a rule unless severe side effects occurred. Dose range was 5–30 mg/day aripiprazole and 2–6 mg risperidone. The dose and type of antipsychotic medication could be changed, at the physician’s discretion, based on clinical efficacy and the profile of side effects during the follow-up period. Initial prescribed medications should be switched to olanzapine (range dose 5–20 mg) due to lack of clinical response at 3 weeks (less than 30% of total BPRS score reduction and CGI ≥ 5) or persisting distressing adverse drug reactions. For patients who fail to reach a clinical response after two anti-psychotic agents, clozapine should be started. However, despite this recommendation, clozapine treatment should not be commenced at clinician’s discretion.

For the present investigation, drug-naïve first episode patients were divided into two groups based on their clinical response after 3 months of antipsychotic treatment according to their BPRS (Lukoff et al., 1986) total scores: the first group included the top 40% of patients who had the best response to treatment (highest absolute decrease of BPRS score) and are referred to as “best-responders”, and the other group included the bottom 40% of patients with the worst response (lower absolute decrease of BPRS score) and are referred to as “worst-responders”.

At 3-month follow-up patients were on: Aripiprazole (7 worst-responders; 3 best-responders), Risperidone (5 worst-responders; 8 best-responders), Olanzapine (3 worst-responders; 4 best-responders).

No statistically significant differences between the two groups (best vs. worst clinical response patients) with respect to percentage of concomitant use of antidepressants, antimuscarinics, hypnotics and benzodiazepines at 3 months were observed (all *p*’s > 0.542). After 6 months follow-up patients were diagnosed as follows, in the group of patients with the best-response to antipsychotic treatment: nine schizophrenia, four schizophreniform disorder, one brief psychotic disorder, and one schizoaffective disorder; in the group of patients with worst-response to antipsychotic treatment: nine schizophrenia, four schizophreniform disorder and two brief psychotic disorder. No significant differences between groups were either found.

### Premorbid and Sociodemographic Variables

Premorbid and sociodemographic information was collected from patients (Table 1), relatives and previous medical records. Age of onset of psychosis was defined as the age when the emergence of the first continuous psychotic symptom occurred. Duration of untreated psychosis (DUP) was defined as the time from the first psychotic symptom to the initiation of antipsychotic drug treatment. Duration of untreated illness (DUI) was defined as the time from the first unspecific symptoms related to psychosis to the initiation of antipsychotic drug treatment.

### Laboratory Assessments

To minimize the effects of diet and technique, blood samples were obtained from fasting subjects from 8:00 to 10:00 a.m. by the same staff, in the same setting. None of the patients had a chronic inflammation or infection, or were taking medication that could influence the results of blood tests.

### RNA Extraction

Total RNA was extracted from blood using the Tempus™ Blood RNA Tube and Tempus™ Spin RNA Isolation Kit (Applied Biosystems, Foster City, CA, USA) following the manufacturer protocols. To define expression profiles, a key factor is that the RNA is intact. To select only high-quality RNA, the RNA Integrity Number (RIN) was characterized with a Bioanalyzer (Agilent Technologies, Santa Clara, CA, USA) and samples with a RIN of at least 7.2 were selected. The selected samples have RINs that range from 8 to 10 with an average of 9.11.

### RNA Next Generation Sequencing

Total RNA was extracted from peripheral whole blood of each individual. The mRNA obtained from blood was sequenced at the Centro Nacional de Análisis Genómico (CNAG) using Illumina HiSeq instruments (San Diego, CA, USA). The mRNA was isolated from the total RNA and was fragmented once transformed into cDNA. Fragments of 300 bp on average were selected to construct the libraries for sequencing. Pair-end sequences of 70 nucleotides for each end were produced.

### Alignment of Reads to the Human Genome Reference

Alignment of the reads was performed in an SLURM HPC server running Tophat 2.0.6 with default options (Trapnell et al., 2009). Tophat aligns RNA-Seq reads to genomes using the Bowtie 2.0.2 alignment program (Langmead et al., 2009), and then analyses the mapping results to identify splice junctions between exons.

### Differential Expression Statistical Analyses

Bedtools 2.17.0 (multicov option; Quinlan and Hall, 2010) was used to count the amount of reads mapped to each gene. The Reference Sequence (RefSeq) gene coordinates were defined using the RefFlat file from the UCSC Genome Bioinformatics Site (as February 28th, 2014). DESeq 1.4 package (Anders and Huber, 2010), setting up fit-only as fitting method, was used to test for differential expression using gene-count data. Two sided Fisher tests were carried out to identify functional enrichment of biological annotations.

### Prediction Method

Gene selection was performed with the implementation of Random Forest method in the Random Forest 4.6-12 package (Breiman, 2001) of R. Expression values of the 130 genes with significantly different expression between the best-responders and worst-responders was used as input with default parameters. Genes with the best Gini were selected for the predictor. It was trained using Logistic regression (Cox, 1958) with the glm function of R 3.2.3 and the calculation of the estimated cross-validation was performed with the cv.glm function of boot package which implements bootstrapping methods (Hinkley, 1988).

## Results

### Differential Gene Expression Between Best-Responders and Worst-Responders Before Treatment

We found 130 genes with significant differential expression between the best-responders and the worst-responders (*P*_adj_ value < 0.01; Supplementary Table S1). These genes were significantly enriched for schizophrenia related genes according to the scientific literature in the Gene Reference into Function (GeneRIF) database (Mitchell et al., 2003). We found 14 schizophrenia differential expression genes between the best-responders and the worst-responders (13.4% observed vs. 6.8% expected; Fisher *P* value = 0.016).

### Differential Gene Expression Between the Best-Responders and the Worst-Responders After Treatment

To obtain more information, we sequenced the transcriptome of the patients after 3 months of treatment with antipsychotics. We defined 219 differential expression genes between the best-responders and the worst-responders after 3 months of medication (Supplementary Table S2). These genes were enriched significantly for schizophrenia (21 genes or 11.3% vs. 6.8%; Fisher *P* value = 0.027). After 3 months of medication with antipsychotics, 6 out of the 14 schizophrenia-annotated genes with differential expression before medication between the best-responders and the worst-responders no longer had differential expression: *VWF*, a glycoprotein with increased levels in plasma of non-medicated patients and in bipolar disorder and schizophrenia compared to control individuals (Yri et al., 2012); *UGT1A1*, a gene with promoter variations in patients with schizophrenia that result in lower serum bilirubin levels (Vitek et al., 2010); *HMOX1*, an enzyme that has anti-inflammatory properties and mediates the first step of heme catabolism involved in the production of carbon monoxide, a putative neurotransmitter, is over-expressed in transgenic mice with schizophrenia-like features (Song et al., 2012); *IL8* (also known as *CXCL8*), a chemokine with altered expression in the dorsolateral prefrontal cortex of individuals with schizophrenia (Fillman et al., 2013); *NTNG2*, a gene with haplotypes associated with schizophrenia and isoform expression significantly different in schizophrenic and control brains (Aoki-Suzuki et al., 2005); and *PTGDS*, a prostaglandin that acts as a neuromodulator as well as a trophic factor in the central nervous system and was studied as a schizophrenia candidate (Ruano et al., 2007) and with reduced mRNA expression in peripheral blood of bipolar disorder patients compared with healthy control subjects (Munkholm et al., 2015). The differential expression genes between the best-responders and the worst-responders after 3 months of medication indicates that 5 out the 11 genes involved in “drug processing” with differential expression before medication still had differential expression and similar expression profile after medication. These five genes are: *GSTM1*, a glutathione S-transferase involved in the detoxification of electrophilic compounds, such as therapeutic drugs, by conjugation with glutathione, with genetic variations that affect the toxicity and efficacy of certain drugs (Li et al., 2013); *THBS1*, an adhesive glycoprotein that mediates cell-to-cell and cell-to-matrix interactions and related to drug resistance (Rath et al., 2006); *PRAME*, an antigen related to cytotoxic drug sensitivity (Kewitz and Staege, 2013); *GSTT1*, a glutathione S-transferase that functions as a drug metabolizing enzyme (Yri et al., 2012); and *SLC22A16*, a solute carrier reported to be involved in drug response (Aouida et al., 2010).

### Differential Gene Expression of the Best-Responders Before and After Treatment and of the Worst-Responders Before and After Treatment

We also compared the transcriptome of the best-responders before and after medication and characterized 176 genes with differential expression (Supplementary Table S3). These genes were also enriched for schizophrenia annotations (9.4% observed vs. 6.7% expected). When we defined the differential expression genes of the worst-responders, before and after medication, we found only 23 genes (Supplementary Table S4) and these were not enriched for schizophrenia annotations (5.2% vs. 6.7%). These data indicate that the individuals that respond worst to treatment have fewer altered genes (a 7.6 fold decrease) in their expression by antipsychotics than the best-responders.

### Predictor Test

**Figure 1:**
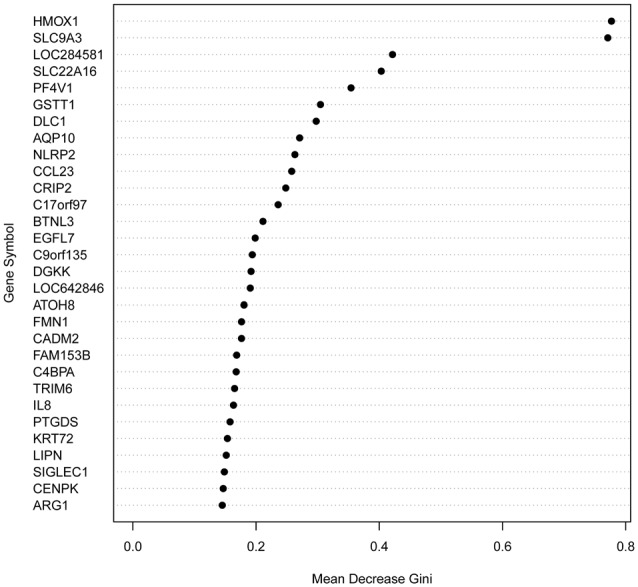
Variable Importance (Gini) for the top 30 predictor genes. Gini variable importance measures reflect the mean decrease in impurity by splits of a given variable in the classification tree, weighted by the proportion of samples reaching that node. A greater “mean decrease Gini” indicates that the gene plays a greater role in partitioning the data into the defined classes.

To generate a predictor test before medication of response to antipsychotics, we analyzed all 130 genes with significantly different expression between the best-responders and the worst-responders using Random Forests, an ensemble learning method for classification and regression, among other tasks, that operates by constructing a multitude of decision trees (Díaz-Uriarte and Alvarez de Andrés, 2006; Koo et al., 2013). Functional analyses of the 30 genes with the highest predictive power (Figure 1; Supplementary Table S5) indicated a significant enrichment of genes related to schizophrenia (29.2% observed vs. 6.9% expected; Fisher *P* value = 0.0009) and bipolar disorder (16.7% vs. 2.6%; Fisher *P* value = 0.003). In the complete list of 130 genes, the enrichment for schizophrenia genes was smaller (13.4%).

**Figure 2:**
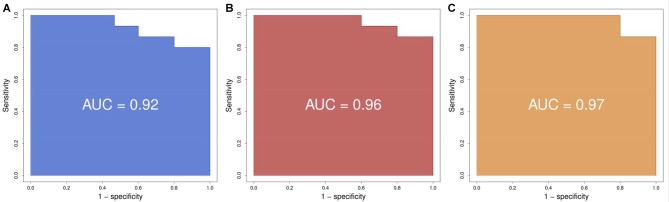
Receiver operating characteristic (ROC) curves. **(A)** Using the best two genes (*HMOX1*, *SCL9A3*) to train the predictor we obtain a ROC with an area under the curve (AUC) of 0.92. **(B)** Using the best three genes (*HMOX1, SCL9A3, SLC22A16*) to train the predictor we obtain a ROC with an AUC of 0.96. **(C)** Using the best four genes (*HMOX1, SCL9A3, SLC22A16, LOC284581*) to train the predictor we obtain a ROC with an AUC of 0.97.

Using logistic regression we defined the area under the curve (*AUC*), representing the combined predictive power of the genes, and a cross-validation estimate of accuracy for the prediction using the first two, three and four genes. We found that the prediction using two genes (*SLC9A3* and *HMOX1*) had an area under the curve of 0.92 and a cross-validation estimate of accuracy of 0.73; with three genes (adding *SLC22A16*) the values were 0.96 and 0.833 respectively; and with four genes (adding *LOC284581*) they were 0.97 and 0.833 respectively (Figure 2). These data indicate that a test with the top four or three of the most predictive genes could be an appropriate choice.

## Discussion

We found a significantly different number of schizophrenia differential expression genes between the groups of best-responders and worst-responders, suggesting that the response to treatment could be due, at least partially, to the fact that the two groups of patients analyzed have different genetic background causing schizophrenia.

Our results indicate that 6 out of the 14 schizophrenia-annotated genes with differential expression before medication between the best and worst-responders had no longer differential expression after 3 months of medication. These six genes are excellent candidates to be the targets used by the drugs to improve the symptoms of schizophrenia. The data show that the group of best-responders has a significant enrichment of schizophrenia-annotated genes with differential expression before and after 3 months of medication. However, we do not observe this significant enrichment of schizophrenia-annotated genes in the group of worst-responders, suggesting that the efficacy of antipsychotics is dependent of the expression profile of the patient before medication and that a predictor could be generated using a gene expression profile of untreated patients.

With the purpose of finding genes that can predict the response to antipsychotics, we characterized 130 genes with differential expression between the best and worst responders before treatment. We found that the 30 genes with the highest predictive power among these 130 genes, had a higher enrichment of schizophrenia-annotated genes, indicating that genes related to schizophrenia tend to have a higher predictive value. This would suggest that schizophrenia genes are involved in the response and that the schizophrenia causative genes tend to be different between the best-responders and the worst-responders.

**Figure 3:**
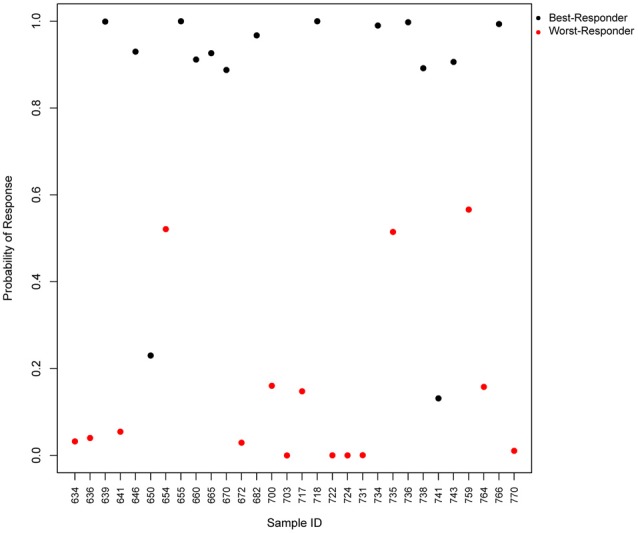
Predicted probability of response to antipsychotics using a 4-gene test. Scatter plot, which represents the predicted probability of response (y-axis) for each input sample (x-axis) in the logistic regression predictor. Red points represent worst-responder patients and black points represent best-responder patients.

We tested the predictive power of the top four predictor genes in our patients, and found that the test would accurately predict 93% of the worst-responders and 87% of the best-responders (Figure 3). The gene with the highest predictive value, *SLC9A3*, is a Na/H exchanger and belongs to several pathways such as the transmembrane transportation of small molecules and the SLC-mediated transmembrane transport that facilitate the movement of a specific substrate either against or following their concentration gradient. Currently, there are novel drugs being developed targeting *SLC9A3* that could be of interest for the field of schizophrenia (Dominguez Rieg et al., 2016). The second best predictor is *HMOX1*, or Heme oxygenase, an essential enzyme in heme catabolism, that cleaves heme to form biliverdin, which is subsequently converted to bilirubin by biliverdin reductase, and carbon monoxide, a putative neurotransmitter. The relationship of this gene with the generation of a neurotransmitter could explain the relationship of his expression with the response to antipsychotics and open the gate to possible medical actions. The previous observations are consistent with the fact that the mouse homologous gene has been related to schizophrenia-like features in transgenic mice, what can facilitate new therapeutics for schizophrenia (Song et al., 2012). *HMOX1* has been also related to drug resistance in acute myeloid leukemia (Zhe et al., 2015). The third predictor, *SLC22A16*, encodes a member of the organic zwitterion transporter protein family which transports carnitine. The encoded protein has also been shown to be involved in the transport of anticancer drugs such as bleomycin (Aouida et al., 2010) and successful treatment has been correlated with the level of activity of this transporter in tumor cells. Variant alleles of SLC22A16 are associated with response and levels of toxicity caused by doxorubicin and cyclophosphamide therapy in the treatment of breast cancer which is consistent with the fact that doxorubicin is a substrate this transporter (Bray et al., 2010). Interestingly, SLC22A16 is located within a schizophrenia susceptibility locus in chromosome 6q (Cao et al., 1997). The fourth gene, *LOC284581*, is not annotated but it maps within a Parkinson’s disease locus of 15.8 Mb (Satake et al., 2009). Interestingly, all three annotated genes are involved in drug resistance, toxicity or transportation and offer new possibilities to generate therapeutic actions for schizophrenia.

A potential limitation of the study is the small sample size (*n* = 30 or *n* = 15 per group). However, we are not aware of any full transcriptome study on drug-naïve patients (not having received a single dose of antipsychotics) that had studied similar or higher number of samples than our study. Besides, our study sample size is larger than in most studies based on RNASeq data (according to the mean of samples reported by the Gene Expression Omnibus database). A possible confounding factor could be the cannabis use that has been reported to enhance negative outcomes such as suicidal risk (Serafini et al., 2012). However, it is unlikely that this factor could affect the results of our study given that only eight patients reported as cannabis users and were equally divided in both groups, four in the best-responders and four in the worst-responders. The data we are presenting here is a first step to generate a simple test (expression levels of a small number of genes) to predict the response to antipsychotics, one of the most prescribed types of drugs worldwide, and to provide a tool to select the antipsychotic expected to generate the best clinical response.

## Author Contributions

JS, CP and BC-F designed the study, analyzed the data and wrote the manuscript; JS and BC-F directed and supervised the research; BC-F performed the clinical analysis; CP and JS performed the bioinformatic work; FR-J provided technical assistance and was responsible for sample managing.

## Conflict of Interest Statement

The authors declare that the research was conducted in the absence of any commercial or financial relationships that could be construed as a potential conflict of interest.

## References

[B1] Alexander G. C. Gallagher S. A. Mascola A. Moloney R. M. Stafford R. S. (2011). Increasing off-label use of antipsychotic medications in the United States, 1995–2008. Pharmacoepidemiol. Drug Saf. 20, 177–184. 10.1002/pds.2082 21254289 PMC3069498

[B2] Anders S. Huber W. (2010). Differential expression analysis for sequence count data. Genome Biol. 11:R106. 10.1186/gb-2010-11-10-r106 20979621 PMC3218662

[B3] Aoki-Suzuki M. Yamada K. Meerabux J. Iwayama-Shigeno Y. Ohba H. Iwamoto K.. (2005). A family-based association study and gene expression analyses of netrin-G1 and -G2 genes in schizophrenia. Biol. Psychiatry 57, 382–393. 10.1016/j.biopsych.2004.11.022 15705354

[B4] Aouida M. Poulin R. Ramotar D. (2010). The human carnitine transporter SLC22A16 mediates high affinity uptake of the anticancer polyamine analogue bleomycin-A5. J. Biol. Chem. 285, 6275–6284. 10.1074/jbc.M109.046151 20037140 PMC2825423

[B5] Bray J. Sludden J. Griffin M. J. Cole M. Verrill M. Jamieson D.. (2010). Influence of pharmacogenetics on response and toxicity in breast cancer patients treated with doxorubicin and cyclophosphamide. Br. J. Cancer 102, 1003–1009. 10.1038/sj.bjc.6605587 20179710 PMC2844036

[B6] Breiman L. (2001). Random forests. Mach. Learn. 45, 5–32. 10.1023/A:1010933404324

[B7] Cao Q. Martinez M. Zhang J. Sanders A. R. Badner J. A. Cravchik A.. (1997). Suggestive evidence for a schizophrenia susceptibility locus on chromosome 6q and a confirmation in an independent series of pedigrees. Genomics 43, 1–8. 10.1006/geno.1997.4815 9226366

[B8] Carton L. Cottencin O. Lapeyre-Mestre M. Geoffroy P. A. Favre J. Simon N.. (2015). Off-label prescribing of antipsychotics in adults, children and elderly individuals: a systematic review of recent prescription trends. Curr. Pharm. Des. 21, 3280–3297. 10.2174/1381612821666150619092903 26088115

[B9] Cox D. R. (1958). The regression-analysis of binary sequences. J. R. Stat. Soc. B Stat. Methodol. 20, 215–242.

[B10] Crespo-Facorro B. Pelayo-Terán J. M. Pérez-Iglesias R. Ramírez-Bonilla M. Martínez-García O. Pardo-García G.. (2007). Predictors of acute treatment response in patients with a first episode of non-affective psychosis: sociodemographics, premorbid and clinical variables. J. Psychiatr. Res. 41, 659–666. 10.1016/j.jpsychires.2006.05.002 16797591

[B11] Crespo-Facorro B. Prieto C. Sainz J. (2015). Schizophrenia gene expression profile reverted to normal levels by antipsychotics. Int. J. Neuropsychopharmacol. 18:pyu066. 10.1093/ijnp/pyu066 25522406 PMC4360232

[B12] Díaz-Uriarte R. Alvarez de Andrés S. (2006). Gene selection and classification of microarray data using random forest. BMC Bioinformatics 7:3. 10.1186/1471-2105-7-3 16398926 PMC1363357

[B13] Dominguez Rieg J. A. de la Mora Chavez S. Rieg T. (2016). Novel developments in differentiating the role of renal and intestinal sodium hydrogen exchanger 3. Am. J. Physiol. Regul. Integr. Comp. Physiol. 311, R1186–R1191. 10.1152/ajpregu.00372.2016 27733387 PMC5256969

[B14] Fillman S. G. Cloonan N. Catts V. S. Miller L. C. Wong J. McCrossin T.. (2013). Increased inflammatory markers identified in the dorsolateral prefrontal cortex of individuals with schizophrenia. Mol. Psychiatry 18, 206–214. 10.1038/mp.2012.110 22869038

[B15] Hinkley D. V. (1988). Bootstrap methods. J. R. Stat. Soc. B Methodol. 50, 321–337.

[B16] Insel T. R. (2010). Rethinking schizophrenia. Nature 468, 187–193. 10.1038/nature09552 21068826

[B17] Kantor E. D. Rehm C. D. Haas J. S. Chan A. T. Giovannucci E. L. (2015). Trends in prescription drug use among adults in the united states from 1999–2012. JAMA 314, 1818–1831. 10.1001/jama.2015.13766 26529160 PMC4752169

[B18] Kewitz S. Staege M. S. (2013). Knock-down of PRAME increases retinoic acid signaling and cytotoxic drug sensitivity of Hodgkin lymphoma cells. PLoS One 8:e55897. 10.1371/journal.pone.0055897 23409080 PMC3569423

[B19] Koo C. L. Liew M. J. Mohamad M. S. Salleh A. H. (2013). A review for detecting gene-gene interactions using machine learning methods in genetic epidemiology. Biomed Res. Int. 2013:432375. 10.1155/2013/432375 24228248 PMC3818807

[B20] Langmead B. Trapnell C. Pop M. Salzberg S. L. (2009). Ultrafast and memory-efficient alignment of short DNA sequences to the human genome. Genome Biol. 10:R25. 10.1186/gb-2009-10-3-r25 19261174 PMC2690996

[B21] Li C. Long J. Hu X. Zhou Y. (2013). GSTM1 and GSTT1 genetic polymorphisms and risk of anti-tuberculosis drug-induced hepatotoxicity: an updated meta-analysis. Eur. J. Clin. Microbiol. Infect. Dis. 32, 859–868. 10.1007/s10096-013-1831-y 23377313

[B22] Lukoff D. Liberman R. P. Nuechterlein K. H. (1986). Symptom monitoring in the rehabilitation of schizophrenic patients. Schizophr. Bull. 12, 578–602. 10.1093/schbul/12.4.578 3810065

[B23] Meltzer H. Y. (1997). Treatment-resistant schizophrenia—the role of clozapine. Curr. Med. Res. Opin. 14, 1–20. 10.1185/03007999709113338 9524789

[B24] Mitchell J. A. Aronson A. R. Mork J. G. Folk L. C. Humphrey S. M. Ward J. M. (2003). Gene indexing: characterization and analysis of NLM’s GeneRIFs. AMIA Annu. Symp. Proc. 2003, 460–464. 14728215 PMC1480312

[B25] Munkholm K. Peijs L. Kessing L. V. Vinberg M. (2015). Reduced mRNA expression of PTGDS in peripheral blood mononuclear cells of rapid-cycling bipolar disorder patients compared with healthy control subjects. Int. J. Neuropsychopharmacol. 18:pyu101. 10.1093/ijnp/pyu101 25522430 PMC4376551

[B26] Pouget J. G. Shams T. A. Tiwari A. K. Muller D. J. (2014). Pharmacogenetics and outcome with antipsychotic drugs. Dialogues Clin. Neurosci. 16, 555–566. 25733959 10.31887/DCNS.2014.16.4/jpouget PMC4336924

[B27] Prata D. Mechelli A. Kapur S. (2014). Clinically meaningful biomarkers for psychosis: a systematic and quantitative review. Neurosci. Biobehav. Rev. 45, 134–141. 10.1016/j.neubiorev.2014.05.010 24877683

[B28] Quinlan A. R. Hall I. M. (2010). BEDTools: a flexible suite of utilities for comparing genomic features. Bioinformatics 26, 841–842. 10.1093/bioinformatics/btq033 20110278 PMC2832824

[B29] Rath G. M. Schneider C. Dedieu S. Rothhut B. Soula-Rothhut M. Ghoneim C.. (2006). The C-terminal CD47/IAP-binding domain of thrombospondin-1 prevents camptothecin- and doxorubicin-induced apoptosis in human thyroid carcinoma cells. Biochim. Biophys. Acta 1763, 1125–1134. 10.1016/j.bbamcr.2006.08.001 16962673

[B30] Roberts R. J. Lohano K. K. El-Mallakh R. S. (2016). Antipsychotics as antidepressants. Asia Pac. Psychiatry 8, 179–188. 10.1111/appy.12186 25963405

[B31] Ruano D. Macedo A. Soares M. J. Valente J. Azevedo M. H. Pato C.. (2007). Family-based and case-control studies reveal no association of lipocalin-type prostaglandin D2 synthase with schizophrenia. Am. J. Med. Genet. B Neuropsychiatr. Genet. 144B, 642–646. 10.1002/ajmg.b.30477 17230501

[B32] Satake W. Nakabayashi Y. Mizuta I. Hirota Y. Ito C. Kubo M.. (2009). Genome-wide association study identifies common variants at four loci as genetic risk factors for Parkinson’s disease. Nat. Genet. 41, 1303–1307. 10.1038/ng.485 19915576

[B33] Serafini G. Pompili M. Innamorati M. Rihmer Z. Sher L. Girardi P. (2012). Can cannabis increase the suicide risk in psychosis? A critical review. Curr. Pharm. Des. 18, 5165–5187. 10.2174/138161212802884663 22716157

[B34] Song W. Zukor H. Lin S. H. Hascalovici J. Liberman A. Tavitian A.. (2012). Schizophrenia-like features in transgenic mice overexpressing human HO-1 in the astrocytic compartment. J. Neurosci. 32, 10841–10853. 10.1523/JNEUROSCI.6469-11.2012 22875919 PMC6621004

[B35] Trapnell C. Pachter L. Salzberg S. L. (2009). TopHat: discovering splice junctions with RNA-Seq. Bioinformatics 25, 1105–1111. 10.1093/bioinformatics/btp120 19289445 PMC2672628

[B36] Vitek L. Novotna M. Lenicek M. Novotny L. Eberova J. Petrásek J.. (2010). Serum bilirubin levels and UGT1A1 promoter variations in patients with schizophrenia. Psychiatry Res. 178, 449–450. 10.1016/j.psychres.2009.12.008 20483464

[B37] Yri O. E. Ekstrøm P. O. Hilden V. Gaudernack G. Liestøl K. Smeland E. B.. (2012). Polymorphisms in genes encoding interleukin-10 and drug metabolizing enzymes GSTP1, GSTT1, GSTA1 and UGT1A1 influence risk and outcome in Hodgkin lymphoma. Leuk. Lymphoma 53, 1934–1944. 10.3109/10428194.2012.682307 22475179

[B38] Zhang J. P. Malhotra A. K. (2011). Pharmacogenetics and antipsychotics: therapeutic efficacy and side effects prediction. Expert Opin. Drug Metab. Toxicol. 7, 9–37. 10.1517/17425255.2011.532787 21162693 PMC3057913

[B39] Zhe N. Wang J. Chen S. Lin X. Chai Q. Zhang Y.. (2015). Heme oxygenase-1 plays a crucial role in chemoresistance in acute myeloid leukemia. Hematology 20, 384–391. 10.1179/1607845414y.0000000212 26218201

