# Supplementary material for: Blood Gene Expression Profile Predicts Response to Antipsychotics
Source: Front Mol Neurosci. 2018 Mar 6;11:73. doi: 10.3389/fnmol.2018.00073 (PMC5845714; doi:10.3389/fnmol.2018.00073)
Supplement: Supplementary file 1 [file Table_1.PDF]

S1 Table. Differential expression between best-responders and worst-responders before antipsychotic medication

geneID	Gene Symbol	Base Mean	Base Mean Best-Responders	Base Mean Worst-Responders	Fold Change	Log2 Fold Change	Pval	Padj
8120	AP3B2	179.55	28.62	330.48	11.55	3.53	2.70E-87	5.23E-83
283692	RP11-752G15.3	149.36	24.40	274.32	11.24	3.49	1.15E-80	1.11E-76
3045	HBD	1812.39	404.31	3220.47	7.97	2.99	9.02E-71	5.82E-67
9381	OTOF	152.34	67.92	236.76	3.49	1.80	3.68E-64	1.78E-60
3429	IFI27	92.35	58.59	126.12	2.15	1.11	3.53E-40	1.14E-36
84873	GPR128	20.06	39.02	1.09	0.03	-5.16	3.07E-40	1.14E-36
10917	BTNL3	352.04	192.80	511.27	2.65	1.41	2.37E-37	6.56E-34
23532	PRAME	15.35	30.01	0.70	0.02	-5.43	7.23E-30	1.75E-26
759	CA1	826.66	335.24	1318.08	3.93	1.98	9.33E-30	2.01E-26
6614	SIGLEC1	1582.03	1289.40	1874.65	1.45	0.54	2.80E-29	5.43E-26
56163	RNF17	20.83	38.40	3.25	0.08	-3.56	1.42E-28	2.51E-25
10964	IFI44L	2952.09	2469.30	3434.87	1.39	0.48	2.20E-28	3.55E-25
722	C4BPA	160.74	108.75	212.73	1.96	0.97	4.46E-26	6.64E-23
9911	TMCC2	1273.06	574.64	1971.47	3.43	1.78	1.37E-24	1.89E-21
100506159	LOC100506159	138.07	79.95	196.19	2.45	1.30	7.41E-22	9.56E-19
144453	BEST3	37.62	6.98	68.26	9.78	3.29	8.91E-22	1.08E-18
55553	SOX6	99.88	32.68	167.09	5.11	2.35	5.37E-21	6.12E-18
10529	NEBL	176.06	232.47	119.65	0.51	-0.96	2.12E-18	2.28E-15
124912	SPACA3	13.48	3.55	23.42	6.61	2.72	4.07E-18	4.15E-15
26807	SNORD43	492.18	670.48	313.88	0.47	-1.09	5.82E-18	5.63E-15
85495	RPPH1	20.33	2.64	38.03	14.42	3.85	7.10E-18	6.55E-15
284581	LOC284581	149.25	91.99	206.51	2.24	1.17	3.85E-16	3.38E-13
2993	GYPA	63.90	23.66	104.14	4.40	2.14	7.99E-16	6.73E-13
10561	IFI44	4047.14	3499.45	4594.83	1.31	0.39	1.54E-15	1.24E-12
219539	YPEL4	142.73	60.16	225.31	3.75	1.91	2.31E-15	1.79E-12

11227	GALNT5	27.71	5.46	49.96	9.14	3.19	3.37E-15	2.51E-12
85462	FHDC1	378.73	167.94	589.53	3.51	1.81	5.21E-14	3.74E-11
2944	GSTM1	266.74	350.45	183.03	0.52	-0.94	6.99E-14	4.83E-11
6521	SLC4A1	3834.18	2379.40	5288.95	2.22	1.15	1.52E-13	1.01E-10
389396	GLYATL3	5.86	11.57	0.15	0.01	-6.27	2.49E-13	1.61E-10
50509	COL5A3	340.85	253.33	428.36	1.69	0.76	1.12E-12	6.99E-10
55225	RAVER2	381.98	489.61	274.34	0.56	-0.84	3.81E-12	2.31E-09
10107	TRIM10	136.70	71.27	202.13	2.84	1.50	7.59E-12	4.45E-09
64478	CSMD1	54.11	37.55	70.67	1.88	0.91	1.05E-11	5.98E-09
55363	HEMGN	773.45	467.37	1079.52	2.31	1.21	1.16E-11	6.43E-09
10900	RUNDC3A	650.84	406.64	895.05	2.20	1.14	2.06E-11	1.11E-08
253012	HEPACAM2	31.80	10.48	53.11	5.07	2.34	3.45E-11	1.81E-08
8444	DYRK3	58.76	24.21	93.31	3.85	1.95	6.00E-11	2.98E-08
51327	AHSP	347.64	213.83	481.46	2.25	1.17	5.95E-11	2.98E-08
57156	TMEM63C	283.12	337.55	228.69	0.68	-0.56	9.70E-11	4.70E-08
5730	PTGDS	929.12	729.70	1128.55	1.55	0.63	2.04E-10	9.61E-08
139189	DGKK	158.05	115.00	201.11	1.75	0.81	2.91E-10	1.34E-07
3488	IGFBP5	9.95	3.49	16.40	4.69	2.23	3.40E-10	1.50E-07
138255	C9orf135	3.53	6.88	0.18	0.03	-5.28	3.41E-10	1.50E-07
6231	RPS26	7889.96	9809.03	5970.90	0.61	-0.72	3.89E-10	1.68E-07
6708	SPTA1	153.70	84.38	223.01	2.64	1.40	4.51E-10	1.86E-07
342184	FMN1	690.54	490.55	890.53	1.82	0.86	4.49E-10	1.86E-07
138307	LCN8	41.55	59.73	23.37	0.39	-1.35	5.18E-10	2.09E-07
55655	NLRP2	372.92	285.58	460.26	1.61	0.69	6.72E-10	2.66E-07
146439	CCDC64B	194.88	239.41	150.35	0.63	-0.67	7.74E-10	3.00E-07
6513	SLC2A1	3167.25	1862.83	4471.68	2.40	1.26	8.37E-10	3.16E-07
54855	FAM46C	4335.70	2771.65	5899.74	2.13	1.09	8.64E-10	3.16E-07
85413	SLC22A16	101.25	134.37	68.12	0.51	-0.98	8.59E-10	3.16E-07
400566	C17orf97	82.80	109.95	55.65	0.51	-0.98	2.22E-09	7.96E-07
84913	ATOH8	129.45	170.86	88.03	0.52	-0.96	2.80E-09	9.86E-07

790952	ESRG	20.96	7.95	33.98	4.27	2.10	3.43E-09	1.19E-06
54892	NCAPG2	537.37	439.20	635.55	1.45	0.53	5.71E-09	1.94E-06
9509	ADAMTS2	94.73	129.28	60.19	0.47	-1.10	6.64E-09	2.22E-06
8938	BAIAP3	1602.60	1868.73	1336.47	0.72	-0.48	6.84E-09	2.25E-06
56603	CYP26B1	33.24	22.21	44.26	1.99	0.99	9.05E-09	2.92E-06
389337	ARHGEF37	45.12	19.91	70.33	3.53	1.82	1.81E-08	5.73E-06
383	ARG1	475.61	349.63	601.59	1.72	0.78	5.06E-08	1.58E-05
2038	EPB42	490.84	351.24	630.45	1.79	0.84	7.35E-08	2.26E-05
266727	MDGA1	520.39	435.74	605.03	1.39	0.47	8.73E-08	2.64E-05
7450	VWF	272.88	311.44	234.31	0.75	-0.41	1.13E-07	3.36E-05
6423	SFRP2	71.69	36.55	106.83	2.92	1.55	1.47E-07	4.30E-05
160364	CLEC12A	9568.99	8072.72	11065.26	1.37	0.45	1.72E-07	4.98E-05
3434	IFIT1	3180.19	3193.88	3166.51	0.99	-0.01	1.94E-07	5.54E-05
165530	CLEC4F	176.50	225.04	127.95	0.57	-0.81	2.08E-07	5.84E-05
10395	DLC1	41.54	60.10	22.98	0.38	-1.39	2.27E-07	6.28E-05
140807	KRT72	514.60	404.45	624.75	1.54	0.63	2.89E-07	7.88E-05
7057	THBS1	2155.78	1848.89	2462.67	1.33	0.41	3.23E-07	8.69E-05
64284	RAB17	8.90	2.85	14.96	5.25	2.39	3.74E-07	9.91E-05
51162	EGFL7	97.37	122.92	71.81	0.58	-0.78	5.24E-07	1.37E-04
6550	SLC9A3	564.98	398.15	731.80	1.84	0.88	5.68E-07	0.00015
4070	TACSTD2	69.68	50.14	89.22	1.78	0.83	5.82E-07	0.00015
148534	TMEM56	107.49	67.18	147.81	2.20	1.14	6.45E-07	0.00016
6563	SLC14A1	718.86	489.90	947.81	1.93	0.95	8.18E-07	0.00020
6368	CCL23	19.79	30.38	9.21	0.30	-1.72	8.99E-07	0.00022
117854	TRIM6	77.44	60.77	94.11	1.55	0.63	9.37E-07	0.00023
3816	KLK1	46.44	57.64	35.23	0.61	-0.71	9.87E-07	0.00023
7138	TNNT1	136.37	95.71	177.03	1.85	0.89	9.85E-07	0.00023
1305	COL13A1	76.00	56.20	95.79	1.70	0.77	1.04E-06	0.00024
1281	COL3A1	4.84	8.84	0.84	0.09	-3.40	1.15E-06	0.00026
219970	GLYATL2	11.46	19.77	3.15	0.16	-2.65	1.29E-06	0.00029

253559	CADM2	21.22	12.08	30.37	2.51	1.33	1.48E-06	0.00033
287	ANK2	196.32	239.50	153.14	0.64	-0.65	1.72E-06	0.00038
91543	RSAD2	2004.32	2151.73	1856.91	0.86	-0.21	1.87E-06	0.00041
3118	HLA-DQA2	428.81	500.75	356.87	0.71	-0.49	2.05E-06	0.00045
10398	MYL9	769.33	889.78	648.88	0.73	-0.46	2.23E-06	0.00048
645843	TMEM14E	10.87	17.52	4.22	0.24	-2.05	2.49E-06	0.00053
54659	UGT1A3	1.65	0.00	3.30	#N/A	#N/A	3.26E-06	0.00069
2952	GSTT1	158.01	115.52	200.50	1.74	0.80	3.54E-06	0.00074
3042	HBM	1017.39	791.33	1243.46	1.57	0.65	4.21E-06	0.00087
7849	PAX8	1475.32	1642.33	1308.32	0.80	-0.33	4.25E-06	0.00087
728577	CNTNAP3B	21.52	32.14	10.90	0.34	-1.56	4.73E-06	0.00095
57596	BEGAIN	190.69	164.86	216.52	1.31	0.39	4.89E-06	0.00098
54658	UGT1A1	1.58	0.00	3.16	#N/A	#N/A	5.28E-06	0.00104
5197	PF4V1	153.20	193.79	112.60	0.58	-0.78	5.33E-06	0.00104
954	ENTPD2	89.39	105.54	73.25	0.69	-0.53	6.23E-06	0.00121
140733	MACROD2	269.64	312.80	226.47	0.72	-0.47	7.61E-06	0.00146
2537	IFI6	2117.51	2050.92	2184.10	1.06	0.09	8.23E-06	0.00155
654433	PAX8-AS1	1197.05	1329.06	1065.05	0.80	-0.32	8.20E-06	0.00155
284751	RP11-290F20.1	1533.19	1351.16	1715.22	1.27	0.34	9.12E-06	0.00170
643418	LIPN	617.26	546.53	687.99	1.26	0.33	1.13E-05	0.00209
928	CD9	880.02	967.64	792.39	0.82	-0.29	1.20E-05	0.00220
55228	PNMAL1	11.20	6.07	16.34	2.69	1.43	1.47E-05	0.00263
202134	FAM153B	113.48	91.43	135.54	1.48	0.57	1.46E-05	0.00263
346171	ZFP57	49.28	60.12	38.45	0.64	-0.64	1.77E-05	0.00314
64105	CENPK	717.36	623.84	810.89	1.30	0.38	2.05E-05	0.00361
642846	LOC642846	254.05	209.62	298.49	1.42	0.51	2.37E-05	0.00414
439996	IFIT1B	274.62	207.22	342.01	1.65	0.72	2.50E-05	0.00432
2213	FCGR2B	1713.15	1898.04	1528.26	0.81	-0.31	2.56E-05	0.00435
387837	CLEC12B	709.07	628.53	789.60	1.26	0.33	2.56E-05	0.00435
1397	CRIP2	478.52	375.84	581.20	1.55	0.63	2.82E-05	0.00473

101927586	RP11-290F20.2	714.29	636.16	792.43	1.25	0.32	2.83E-05	0.00473
89872	AQP10	80.19	98.55	61.83	0.63	-0.67	2.93E-05	0.00485
3162	HMOX1	2923.47	3437.52	2409.42	0.70	-0.51	3.43E-05	0.00562
440068	CARD17	159.68	135.81	183.55	1.35	0.43	3.49E-05	0.00569
100272216	LOC100272216	457.54	396.96	518.12	1.31	0.38	4.22E-05	0.00682
54600	UGT1A9	3.74	1.07	6.42	6.01	2.59	4.40E-05	0.00704
420	ART4	7.01	1.43	12.59	8.83	3.14	4.50E-05	0.00707
5473	PPBP	3554.04	4097.65	3010.43	0.73	-0.44	4.53E-05	0.00707
84628	NTNG2	4548.39	3892.14	5204.64	1.34	0.42	4.49E-05	0.00707
440836	ODF3B	1892.18	1723.22	2061.14	1.20	0.26	4.58E-05	0.00710
2740	GLP1R	14.09	8.70	19.47	2.24	1.16	5.33E-05	0.00819
3576	IL8	489.24	387.59	590.88	1.52	0.61	5.38E-05	0.00820
6517	SLC2A4	7.04	1.93	12.15	6.30	2.65	5.57E-05	0.00843
339975	RP11-138B4.1	24.95	33.00	16.90	0.51	-0.97	5.72E-05	0.00859
284194	LGALS9B	79.57	64.45	94.69	1.47	0.56	5.97E-05	0.00890

Headers of the Table	
geneID	Gene Identification
Gene Symbol	Official Symbol
Base Mean	Mean normalized counts, averaged over all samples from both conditions
Base Mean Best-Responders	Mean normalized counts from condition A
Base Mean Worst-Responders	Mean normalized counts from condition B
Fold Change	Fold change from condition A to B
Log2 Fold Change	The logarithm, to basis 2, of the fold change
P value	P value for the statistical significance of this change
Padj	P value adjusted for multiple testing with the Benjamini-Hochberg procedure, which controls false discovery rate
