# Supplementary material for: Blood Gene Expression Profile Predicts Response to Antipsychotics
Source: Front Mol Neurosci. 2018 Mar 6;11:73. doi: 10.3389/fnmol.2018.00073 (PMC5845714; doi:10.3389/fnmol.2018.00073)
Supplement: Supplementary file 2 [file Table_2.DOCX]

| **S2 Table. Differential expression between best-responders and worst-responders after 3 months of antipsychotic medication** | | | | | | | | |
| --- | --- | --- | --- | --- | --- | --- | --- | --- |
| geneID | Gene Symbol | Base Mean | Base Mean Best-Responders | Base Mean Worst- Responders | Fold Change | Log2 Fold Change | Pval | Padj |
| 100462981 | MTRNR2L2 | 552.17 | 123.89 | 980.46 | 7.91 | 2.98 | 6.64E-138 | 1.28E-133 |
| 8120 | AP3B2 | 129.22 | 28.24 | 230.20 | 8.15 | 3.03 | 1.06E-87 | 1.02E-83 |
| 283692 | RP11-752G15.3 | 109.64 | 24.55 | 194.74 | 7.93 | 2.99 | 2.78E-79 | 1.79E-75 |
| 10562 | OLFM4 | 474.08 | 750.34 | 197.82 | 0.26 | -1.92 | 3.36E-59 | 1.62E-55 |
| 84873 | GPR128 | 19.65 | 38.68 | 0.62 | 0.02 | -5.96 | 8.09E-51 | 3.12E-47 |
| 3045 | HBD | 1029.61 | 539.01 | 1520.22 | 2.82 | 1.50 | 3.69E-43 | 1.19E-39 |
| 56163 | RNF17 | 19.42 | 37.26 | 1.57 | 0.04 | -4.57 | 2.06E-41 | 5.69E-38 |
| 10917 | BTNL3 | 320.46 | 168.86 | 472.06 | 2.80 | 1.48 | 7.74E-40 | 1.87E-36 |
| 4317 | MMP8 | 470.84 | 703.03 | 238.64 | 0.34 | -1.56 | 7.00E-36 | 1.50E-32 |
| 23532 | PRAME | 18.62 | 35.32 | 1.92 | 0.05 | -4.20 | 9.30E-35 | 1.79E-31 |
| 4057 | LTF | 3926.52 | 5762.89 | 2090.14 | 0.36 | -1.46 | 2.52E-34 | 4.43E-31 |
| 91543 | RSAD2 | 1543.30 | 885.85 | 2200.75 | 2.48 | 1.31 | 1.74E-33 | 2.81E-30 |
| 722 | C4BPA | 214.41 | 97.75 | 331.07 | 3.39 | 1.76 | 8.47E-33 | 1.26E-29 |
| 212 | ALAS2 | 5103.16 | 6871.26 | 3335.07 | 0.49 | -1.04 | 4.01E-28 | 5.52E-25 |
| 85462 | FHDC1 | 274.44 | 163.89 | 384.99 | 2.35 | 1.23 | 4.48E-28 | 5.76E-25 |
| 3934 | LCN2 | 1072.81 | 1532.41 | 613.22 | 0.40 | -1.32 | 1.45E-27 | 1.75E-24 |
| 10964 | IFI44L | 2221.93 | 1379.96 | 3063.90 | 2.22 | 1.15 | 5.31E-25 | 6.03E-22 |
| 1088 | CEACAM8 | 625.62 | 883.95 | 367.29 | 0.42 | -1.27 | 3.21E-24 | 3.44E-21 |
| 221687 | RNF182 | 75.43 | 113.18 | 37.68 | 0.33 | -1.59 | 1.54E-23 | 1.57E-20 |
| 284581 | LOC284581 | 162.60 | 93.73 | 231.48 | 2.47 | 1.30 | 1.33E-22 | 1.28E-19 |
| 55553 | SOX6 | 63.76 | 32.50 | 95.03 | 2.92 | 1.55 | 5.77E-22 | 5.31E-19 |
| 3434 | IFIT1 | 2548.79 | 1676.53 | 3421.05 | 2.04 | 1.03 | 1.30E-21 | 1.14E-18 |
| 10321 | CRISP3 | 299.26 | 421.07 | 177.44 | 0.42 | -1.25 | 1.81E-21 | 1.52E-18 |
| 10561 | IFI44 | 3516.25 | 2318.88 | 4713.62 | 2.03 | 1.02 | 1.92E-21 | 1.54E-18 |
| 1719 | DHFR | 1191.43 | 775.34 | 1607.53 | 2.07 | 1.05 | 2.02E-21 | 1.56E-18 |
| 6614 | SIGLEC1 | 1087.26 | 706.54 | 1467.97 | 2.08 | 1.05 | 8.35E-21 | 6.20E-18 |
| 154664 | ABCA13 | 308.12 | 429.63 | 186.61 | 0.43 | -1.20 | 4.50E-20 | 3.22E-17 |
| 100463486 | MTRNR2L8 | 161.91 | 95.55 | 228.26 | 2.39 | 1.26 | 9.92E-20 | 6.84E-17 |
| 9911 | TMCC2 | 901.96 | 633.16 | 1170.75 | 1.85 | 0.89 | 1.64E-19 | 1.09E-16 |
| 3437 | IFIT3 | 5156.78 | 3553.56 | 6759.99 | 1.90 | 0.93 | 1.75E-19 | 1.13E-16 |
| 7057 | THBS1 | 2540.71 | 1725.05 | 3356.38 | 1.95 | 0.96 | 7.26E-19 | 4.52E-16 |
| 138307 | LCN8 | 37.43 | 58.14 | 16.72 | 0.29 | -1.80 | 3.11E-18 | 1.87E-15 |
| 9172 | MYOM2 | 768.61 | 943.02 | 594.19 | 0.63 | -0.67 | 4.80E-18 | 2.80E-15 |
| 219539 | YPEL4 | 94.39 | 57.90 | 130.89 | 2.26 | 1.18 | 1.52E-17 | 8.65E-15 |
| 820 | CAMP | 627.74 | 838.84 | 416.63 | 0.50 | -1.01 | 1.28E-16 | 7.04E-14 |
| 1667 | DEFA1 | 2085.15 | 2693.95 | 1476.34 | 0.55 | -0.87 | 2.82E-16 | 1.43E-13 |
| 728358 | DEFA1B | 2085.15 | 2693.95 | 1476.34 | 0.55 | -0.87 | 2.82E-16 | 1.43E-13 |
| 1668 | DEFA3 | 2085.15 | 2693.95 | 1476.34 | 0.55 | -0.87 | 2.82E-16 | 1.43E-13 |
| 100423062 | IGLL5 | 2409.85 | 1690.79 | 3128.91 | 1.85 | 0.89 | 3.46E-16 | 1.71E-13 |
| 2537 | IFI6 | 1806.34 | 1284.00 | 2328.68 | 1.81 | 0.86 | 4.40E-16 | 2.12E-13 |
| 3512 | IGJ | 4098.29 | 2877.33 | 5319.26 | 1.85 | 0.89 | 5.11E-16 | 2.40E-13 |
| 2944 | GSTM1 | 218.87 | 290.20 | 147.54 | 0.51 | -0.98 | 4.58E-15 | 2.11E-12 |
| 50509 | COL5A3 | 335.15 | 232.24 | 438.05 | 1.89 | 0.92 | 7.73E-15 | 3.47E-12 |
| 759 | CA1 | 520.05 | 372.58 | 667.52 | 1.79 | 0.84 | 8.92E-15 | 3.91E-12 |
| 4680 | CEACAM6 | 451.75 | 596.20 | 307.30 | 0.52 | -0.96 | 6.47E-14 | 2.77E-11 |
| 4973 | OLR1 | 97.07 | 136.85 | 57.30 | 0.42 | -1.26 | 6.95E-14 | 2.92E-11 |
| 100506159 | LOC100506159 | 124.08 | 79.94 | 168.22 | 2.10 | 1.07 | 9.23E-14 | 3.79E-11 |
| 6708 | SPTA1 | 126.50 | 86.02 | 166.99 | 1.94 | 0.96 | 3.40E-13 | 1.37E-10 |
| 85495 | RPPH1 | 11.79 | 3.52 | 20.06 | 5.70 | 2.51 | 4.22E-13 | 1.66E-10 |
| 9636 | ISG15 | 1259.99 | 940.83 | 1579.14 | 1.68 | 0.75 | 4.57E-13 | 1.76E-10 |
| 710 | SERPING1 | 631.34 | 466.82 | 795.86 | 1.70 | 0.77 | 6.75E-13 | 2.55E-10 |
| 389396 | GLYATL3 | 5.19 | 10.16 | 0.23 | 0.02 | -5.48 | 9.30E-13 | 3.45E-10 |
| 7280 | TUBB2A | 100.49 | 136.34 | 64.64 | 0.47 | -1.08 | 9.85E-13 | 3.59E-10 |
| 129607 | CMPK2 | 899.34 | 661.60 | 1137.07 | 1.72 | 0.78 | 1.13E-12 | 4.03E-10 |
| 4599 | MX1 | 5724.84 | 4210.13 | 7239.54 | 1.72 | 0.78 | 1.47E-12 | 5.17E-10 |
| 54855 | FAM46C | 2993.80 | 2295.02 | 3692.59 | 1.61 | 0.69 | 2.53E-12 | 8.73E-10 |
| 6513 | SLC2A1 | 2274.59 | 1762.40 | 2786.78 | 1.58 | 0.66 | 2.68E-12 | 8.91E-10 |
| 56603 | CYP26B1 | 23.63 | 12.77 | 34.48 | 2.70 | 1.43 | 2.65E-12 | 8.91E-10 |
| 165530 | CLEC4F | 166.84 | 210.72 | 122.96 | 0.58 | -0.78 | 2.83E-12 | 9.26E-10 |
| 284751 | RP11-290F20.1 | 1461.11 | 1140.12 | 1782.10 | 1.56 | 0.64 | 3.29E-12 | 1.06E-09 |
| 101927586 | RP11-290F20.2 | 659.11 | 510.32 | 807.90 | 1.58 | 0.66 | 3.34E-12 | 1.06E-09 |
| 5657 | PRTN3 | 73.35 | 103.59 | 43.12 | 0.42 | -1.26 | 3.65E-12 | 1.14E-09 |
| 55225 | RAVER2 | 349.04 | 453.32 | 244.76 | 0.54 | -0.89 | 4.50E-12 | 1.38E-09 |
| 144453 | BEST3 | 18.10 | 8.33 | 27.86 | 3.35 | 1.74 | 4.99E-12 | 1.50E-09 |
| 6285 | S100B | 261.52 | 336.31 | 186.73 | 0.56 | -0.85 | 5.26E-12 | 1.56E-09 |
| 343171 | OR2W3 | 229.33 | 291.40 | 167.25 | 0.57 | -0.80 | 6.23E-12 | 1.82E-09 |
| 51191 | HERC5 | 1417.41 | 1053.58 | 1781.25 | 1.69 | 0.76 | 6.37E-12 | 1.84E-09 |
| 4940 | OAS3 | 4833.10 | 3637.70 | 6028.49 | 1.66 | 0.73 | 6.61E-12 | 1.88E-09 |
| 400566 | C17orf97 | 84.51 | 111.47 | 57.54 | 0.52 | -0.95 | 2.88E-11 | 8.05E-09 |
| 11227 | GALNT5 | 13.23 | 5.15 | 21.31 | 4.14 | 2.05 | 4.40E-11 | 1.21E-08 |
| 6231 | RPS26 | 6784.85 | 8247.69 | 5322.00 | 0.65 | -0.63 | 5.22E-11 | 1.42E-08 |
| 389337 | ARHGEF37 | 36.59 | 21.92 | 51.27 | 2.34 | 1.23 | 5.61E-11 | 1.50E-08 |
| 81788 | NUAK2 | 5971.74 | 4517.00 | 7426.48 | 1.64 | 0.72 | 6.08E-11 | 1.61E-08 |
| 1669 | DEFA4 | 443.13 | 565.57 | 320.69 | 0.57 | -0.82 | 8.39E-11 | 2.19E-08 |
| 731424 | RP11-701P16.5 | 260.18 | 188.94 | 331.41 | 1.75 | 0.81 | 1.00E-10 | 2.58E-08 |
| 2731 | GLDC | 48.30 | 29.56 | 67.04 | 2.27 | 1.18 | 2.87E-10 | 7.29E-08 |
| 3848 | KRT1 | 384.34 | 453.97 | 314.71 | 0.69 | -0.53 | 3.11E-10 | 7.80E-08 |
| 2993 | GYPA | 44.39 | 28.29 | 60.49 | 2.14 | 1.10 | 4.84E-10 | 1.20E-07 |
| 8991 | SELENBP1 | 680.88 | 818.83 | 542.92 | 0.66 | -0.59 | 8.02E-10 | 1.96E-07 |
| 3433 | IFIT2 | 10039.94 | 7870.09 | 12209.80 | 1.55 | 0.63 | 1.21E-09 | 2.92E-07 |
| 8444 | DYRK3 | 34.20 | 21.34 | 47.07 | 2.21 | 1.14 | 1.86E-09 | 4.43E-07 |
| 57094 | CPA6 | 113.84 | 145.61 | 82.07 | 0.56 | -0.83 | 2.39E-09 | 5.64E-07 |
| 6550 | SLC9A3 | 551.54 | 417.38 | 685.70 | 1.64 | 0.72 | 2.68E-09 | 6.23E-07 |
| 55384 | MEG3 | 101.25 | 125.93 | 76.58 | 0.61 | -0.72 | 3.57E-09 | 8.11E-07 |
| 608 | TNFRSF17 | 129.48 | 92.01 | 166.96 | 1.81 | 0.86 | 3.57E-09 | 8.11E-07 |
| 1740 | DLG2 | 5191.39 | 6346.07 | 4036.71 | 0.64 | -0.65 | 4.12E-09 | 9.25E-07 |
| 3240 | HP | 215.96 | 272.32 | 159.60 | 0.59 | -0.77 | 5.67E-09 | 1.26E-06 |
| 3772 | KCNJ15 | 10831.46 | 8493.90 | 13169.03 | 1.55 | 0.63 | 9.04E-09 | 1.97E-06 |
| 339975 | RP11-138B4.1 | 23.80 | 33.78 | 13.81 | 0.41 | -1.29 | 9.07E-09 | 1.97E-06 |
| 10410 | IFITM3 | 5051.19 | 3975.00 | 6127.37 | 1.54 | 0.62 | 9.39E-09 | 2.01E-06 |
| 5197 | PF4V1 | 129.67 | 165.97 | 93.36 | 0.56 | -0.83 | 1.15E-08 | 2.44E-06 |
| 81796 | SLCO5A1 | 19248.31 | 23465.55 | 15031.07 | 0.64 | -0.64 | 1.21E-08 | 2.53E-06 |
| 116369 | SLC26A8 | 711.30 | 541.91 | 880.69 | 1.63 | 0.70 | 1.24E-08 | 2.56E-06 |
| 91947 | ARRDC4 | 1358.72 | 1065.05 | 1652.40 | 1.55 | 0.63 | 1.77E-08 | 3.63E-06 |
| 164045 | HFM1 | 33832.51 | 41213.36 | 26451.67 | 0.64 | -0.64 | 1.79E-08 | 3.63E-06 |
| 100130889 | PSORS1C3 | 28.31 | 17.26 | 39.37 | 2.28 | 1.19 | 1.97E-08 | 3.96E-06 |
| 55363 | HEMGN | 612.19 | 490.07 | 734.30 | 1.50 | 0.58 | 2.09E-08 | 4.17E-06 |
| 5266 | PI3 | 847.57 | 691.84 | 1003.30 | 1.45 | 0.54 | 3.04E-08 | 5.98E-06 |
| 6097 | RORC | 383.74 | 308.60 | 458.87 | 1.49 | 0.57 | 3.31E-08 | 6.44E-06 |
| 138255 | C9orf135 | 3.13 | 5.93 | 0.33 | 0.06 | -4.18 | 5.96E-08 | 1.15E-05 |
| 4353 | MPO | 836.06 | 1025.05 | 647.07 | 0.63 | -0.66 | 7.62E-08 | 1.46E-05 |
| 671 | BPI | 939.24 | 1150.00 | 728.49 | 0.63 | -0.66 | 8.35E-08 | 1.58E-05 |
| 2952 | GSTT1 | 154.68 | 117.07 | 192.29 | 1.64 | 0.72 | 8.47E-08 | 1.59E-05 |
| 10529 | NEBL | 184.80 | 231.70 | 137.91 | 0.60 | -0.75 | 1.15E-07 | 2.14E-05 |
| 5013 | OTX1 | 107.62 | 79.77 | 135.47 | 1.70 | 0.76 | 1.36E-07 | 2.50E-05 |
| 81567 | TXNDC5 | 4622.38 | 3693.53 | 5551.23 | 1.50 | 0.59 | 1.56E-07 | 2.84E-05 |
| 25893 | TRIM58 | 3575.84 | 4203.39 | 2948.28 | 0.70 | -0.51 | 1.94E-07 | 3.51E-05 |
| 6947 | TCN1 | 304.09 | 370.23 | 237.94 | 0.64 | -0.64 | 2.04E-07 | 3.62E-05 |
| 253012 | HEPACAM2 | 19.22 | 11.21 | 27.22 | 2.43 | 1.28 | 2.03E-07 | 3.62E-05 |
| 7850 | IL1R2 | 3233.76 | 2626.24 | 3841.28 | 1.46 | 0.55 | 2.12E-07 | 3.72E-05 |
| 9623 | TCL1B | 38.26 | 51.09 | 25.43 | 0.50 | -1.01 | 2.14E-07 | 3.73E-05 |
| 51237 | MZB1 | 549.74 | 440.95 | 658.53 | 1.49 | 0.58 | 3.04E-07 | 5.23E-05 |
| 1511 | CTSG | 131.57 | 166.48 | 96.66 | 0.58 | -0.78 | 3.09E-07 | 5.27E-05 |
| 6286 | S100P | 2083.80 | 1703.83 | 2463.76 | 1.45 | 0.53 | 3.16E-07 | 5.34E-05 |
| 342184 | FMN1 | 606.97 | 472.63 | 741.32 | 1.57 | 0.65 | 3.40E-07 | 5.70E-05 |
| 7173 | TPO | 10.90 | 5.22 | 16.58 | 3.18 | 1.67 | 3.45E-07 | 5.74E-05 |
| 100133941 | CD24 | 1465.52 | 1746.78 | 1184.26 | 0.68 | -0.56 | 4.26E-07 | 7.02E-05 |
| 64284 | RAB17 | 7.24 | 2.42 | 12.05 | 4.97 | 2.31 | 4.68E-07 | 7.65E-05 |
| 56729 | RETN | 179.35 | 217.24 | 141.47 | 0.65 | -0.62 | 5.12E-07 | 8.30E-05 |
| 6037 | RNASE3 | 193.44 | 239.76 | 147.12 | 0.61 | -0.70 | 5.48E-07 | 8.82E-05 |
| 64478 | CSMD1 | 56.81 | 43.35 | 70.27 | 1.62 | 0.70 | 6.04E-07 | 9.63E-05 |
| 160364 | CLEC12A | 9225.02 | 7419.87 | 11030.17 | 1.49 | 0.57 | 6.23E-07 | 9.84E-05 |
| 83869 | TTTY14 | 1493.66 | 1775.87 | 1211.44 | 0.68 | -0.55 | 6.27E-07 | 9.84E-05 |
| 3627 | CXCL10 | 85.62 | 66.02 | 105.22 | 1.59 | 0.67 | 7.61E-07 | 0.00012 |
| 83999 | KREMEN1 | 1566.49 | 1266.54 | 1866.44 | 1.47 | 0.56 | 7.72E-07 | 0.00012 |
| 383 | ARG1 | 511.83 | 407.42 | 616.23 | 1.51 | 0.60 | 7.90E-07 | 0.00012 |
| 3624 | INHBA | 8.81 | 13.98 | 3.64 | 0.26 | -1.94 | 8.26E-07 | 0.00013 |
| 85413 | SLC22A16 | 90.23 | 114.48 | 65.98 | 0.58 | -0.79 | 8.71E-07 | 0.00013 |
| 5909 | RAP1GAP | 60.30 | 74.07 | 46.54 | 0.63 | -0.67 | 9.39E-07 | 0.00014 |
| 26577 | PCOLCE2 | 17.40 | 25.31 | 9.49 | 0.37 | -1.42 | 9.88E-07 | 0.00015 |
| 283120 | H19 | 32.96 | 44.01 | 21.91 | 0.50 | -1.01 | 1.06E-06 | 0.00016 |
| 10911 | UTS2 | 58.20 | 46.76 | 69.63 | 1.49 | 0.57 | 1.08E-06 | 0.00016 |
| 9828 | ARHGEF17 | 120.98 | 91.33 | 150.64 | 1.65 | 0.72 | 1.11E-06 | 0.00016 |
| 419 | ART3 | 94.70 | 74.08 | 115.32 | 1.56 | 0.64 | 1.13E-06 | 0.00016 |
| 3823 | KLRC3 | 695.43 | 835.56 | 555.31 | 0.66 | -0.59 | 1.24E-06 | 0.00018 |
| 6622 | SNCA | 2073.67 | 2403.36 | 1743.98 | 0.73 | -0.46 | 1.41E-06 | 0.00020 |
| 1308 | COL17A1 | 53.50 | 69.91 | 37.09 | 0.53 | -0.91 | 1.51E-06 | 0.00021 |
| 2078 | ERG | 76.99 | 97.88 | 56.10 | 0.57 | -0.80 | 1.53E-06 | 0.00021 |
| 2999 | GZMH | 2177.16 | 2580.02 | 1774.30 | 0.69 | -0.54 | 1.83E-06 | 0.00025 |
| 387755 | INSC | 80.87 | 58.68 | 103.06 | 1.76 | 0.81 | 2.32E-06 | 0.00032 |
| 57282 | SLC4A10 | 516.94 | 434.44 | 599.43 | 1.38 | 0.46 | 3.02E-06 | 0.00041 |
| 287 | ANK2 | 177.59 | 210.13 | 145.06 | 0.69 | -0.53 | 3.34E-06 | 0.00045 |
| 114132 | SIGLEC11 | 70.79 | 88.88 | 52.70 | 0.59 | -0.75 | 3.45E-06 | 0.00047 |
| 10395 | DLC1 | 34.35 | 46.49 | 22.22 | 0.48 | -1.07 | 3.72E-06 | 0.00050 |
| 6783 | SULT1E1 | 17.60 | 11.01 | 24.18 | 2.20 | 1.14 | 3.81E-06 | 0.00051 |
| 140733 | MACROD2 | 237.94 | 279.11 | 196.78 | 0.71 | -0.50 | 4.04E-06 | 0.00053 |
| 4318 | MMP9 | 4649.16 | 3756.30 | 5542.02 | 1.48 | 0.56 | 4.68E-06 | 0.00061 |
| 54892 | NCAPG2 | 484.67 | 402.83 | 566.51 | 1.41 | 0.49 | 5.06E-06 | 0.00066 |
| 5159 | PDGFRB | 312.18 | 373.38 | 250.99 | 0.67 | -0.57 | 5.36E-06 | 0.00069 |
| 9586 | CREB5 | 11137.40 | 9193.61 | 13081.20 | 1.42 | 0.51 | 5.70E-06 | 0.00073 |
| 253559 | CADM2 | 20.82 | 11.53 | 30.10 | 2.61 | 1.38 | 6.01E-06 | 0.00076 |
| 3092 | HIP1 | 3686.97 | 3053.35 | 4320.59 | 1.42 | 0.50 | 6.00E-06 | 0.00076 |
| 55228 | PNMAL1 | 10.10 | 4.83 | 15.36 | 3.18 | 1.67 | 6.31E-06 | 0.00080 |
| 26807 | SNORD43 | 396.63 | 473.95 | 319.31 | 0.67 | -0.57 | 6.74E-06 | 0.00085 |
| 6368 | CCL23 | 21.83 | 29.53 | 14.13 | 0.48 | -1.06 | 7.31E-06 | 0.00091 |
| 51326 | ARL17A | 312.68 | 260.16 | 365.20 | 1.40 | 0.49 | 7.40E-06 | 0.00091 |
| 100506084 | ARL17B | 312.47 | 259.98 | 364.95 | 1.40 | 0.49 | 7.38E-06 | 0.00091 |
| 1832 | DSP | 181.82 | 219.05 | 144.59 | 0.66 | -0.60 | 7.56E-06 | 0.00092 |
| 5359 | PLSCR1 | 2771.05 | 2306.08 | 3236.03 | 1.40 | 0.49 | 8.09E-06 | 0.00098 |
| 1291 | COL6A1 | 136.93 | 110.17 | 163.68 | 1.49 | 0.57 | 8.75E-06 | 0.00105 |
| 10124 | ARL4A | 348.82 | 291.87 | 405.77 | 1.39 | 0.48 | 8.86E-06 | 0.00106 |
| 92304 | SCGB3A1 | 40.51 | 29.41 | 51.61 | 1.75 | 0.81 | 8.93E-06 | 0.00106 |
| 26873 | OPLAH | 278.40 | 226.74 | 330.06 | 1.46 | 0.54 | 9.04E-06 | 0.00107 |
| 644248 | RP11-67M1.1 | 329.81 | 384.84 | 274.79 | 0.71 | -0.49 | 9.54E-06 | 0.00112 |
| 116071 | BATF2 | 226.21 | 187.96 | 264.46 | 1.41 | 0.49 | 9.68E-06 | 0.00113 |
| 7138 | TNNT1 | 116.95 | 89.84 | 144.07 | 1.60 | 0.68 | 1.05E-05 | 0.00122 |
| 9884 | LRRC37A | 201.81 | 165.72 | 237.91 | 1.44 | 0.52 | 1.14E-05 | 0.00131 |
| 9289 | GPR56 | 5593.89 | 6456.75 | 4731.04 | 0.73 | -0.45 | 1.17E-05 | 0.00134 |
| 57801 | HES4 | 66.94 | 82.07 | 51.81 | 0.63 | -0.66 | 1.41E-05 | 0.00161 |
| 60675 | PROK2 | 6143.39 | 5068.81 | 7217.98 | 1.42 | 0.51 | 1.52E-05 | 0.00172 |
| 391322 | LOC391322 | 101.39 | 80.22 | 122.57 | 1.53 | 0.61 | 1.57E-05 | 0.00177 |
| 604 | BCL6 | 28755.37 | 24065.79 | 33444.95 | 1.39 | 0.47 | 1.63E-05 | 0.00183 |
| 84913 | ATOH8 | 110.77 | 136.68 | 84.86 | 0.62 | -0.69 | 1.67E-05 | 0.00186 |
| 140807 | KRT72 | 573.13 | 487.69 | 658.57 | 1.35 | 0.43 | 1.79E-05 | 0.00198 |
| 7849 | PAX8 | 1429.06 | 1598.50 | 1259.62 | 0.79 | -0.34 | 1.80E-05 | 0.00199 |
| 100526836 | BLOC1S5-TXNDC5 | 5509.08 | 4603.17 | 6414.99 | 1.39 | 0.48 | 2.07E-05 | 0.00227 |
| 642846 | LOC642846 | 254.36 | 207.04 | 301.68 | 1.46 | 0.54 | 2.21E-05 | 0.00240 |
| 1991 | ELANE | 216.55 | 263.80 | 169.29 | 0.64 | -0.64 | 2.22E-05 | 0.00240 |
| 94031 | HTRA3 | 28.52 | 38.65 | 18.39 | 0.48 | -1.07 | 2.36E-05 | 0.00254 |
| 3821 | KLRC1 | 296.33 | 354.41 | 238.24 | 0.67 | -0.57 | 2.49E-05 | 0.00267 |
| 1397 | CRIP2 | 363.20 | 305.60 | 420.80 | 1.38 | 0.46 | 2.56E-05 | 0.00273 |
| 125058 | TBC1D16 | 240.83 | 196.08 | 285.58 | 1.46 | 0.54 | 2.59E-05 | 0.00274 |
| 654433 | PAX8-AS1 | 1162.49 | 1296.90 | 1028.07 | 0.79 | -0.34 | 3.22E-05 | 0.00340 |
| 80310 | PDGFD | 351.57 | 410.73 | 292.41 | 0.71 | -0.49 | 3.49E-05 | 0.00366 |
| 29065 | ASAP1-IT1 | 7.52 | 10.79 | 4.26 | 0.39 | -1.34 | 3.55E-05 | 0.00370 |
| 399697 | CTXN2 | 60.55 | 76.28 | 44.81 | 0.59 | -0.77 | 3.66E-05 | 0.00380 |
| 200315 | APOBEC3A | 6632.11 | 5649.44 | 7614.78 | 1.35 | 0.43 | 3.71E-05 | 0.00383 |
| 6320 | CLEC11A | 150.08 | 178.96 | 121.21 | 0.68 | -0.56 | 4.01E-05 | 0.00411 |
| 440068 | CARD17 | 142.31 | 113.57 | 171.04 | 1.51 | 0.59 | 4.05E-05 | 0.00414 |
| 566 | AZU1 | 278.65 | 335.16 | 222.15 | 0.66 | -0.59 | 4.39E-05 | 0.00446 |
| 619207 | SCART1 | 566.81 | 498.63 | 634.99 | 1.27 | 0.35 | 4.85E-05 | 0.00490 |
| 8843 | HCAR3 | 2614.62 | 2206.75 | 3022.49 | 1.37 | 0.45 | 4.97E-05 | 0.00500 |
| 6563 | SLC14A1 | 646.09 | 547.28 | 744.90 | 1.36 | 0.44 | 5.38E-05 | 0.00536 |
| 875 | CBS | 179.64 | 147.64 | 211.64 | 1.43 | 0.52 | 5.39E-05 | 0.00536 |
| 84418 | CYSTM1 | 746.87 | 625.78 | 867.96 | 1.39 | 0.47 | 5.45E-05 | 0.00539 |
| 83416 | FCRL5 | 1496.00 | 1231.46 | 1760.55 | 1.43 | 0.52 | 5.53E-05 | 0.00544 |
| 100913187 | APOBEC3A_B | 7114.94 | 6076.24 | 8153.63 | 1.34 | 0.42 | 5.57E-05 | 0.00546 |
| 1870 | E2F2 | 606.41 | 519.34 | 693.48 | 1.34 | 0.42 | 5.97E-05 | 0.00579 |
| 3084 | NRG1 | 226.57 | 190.38 | 262.77 | 1.38 | 0.46 | 5.94E-05 | 0.00579 |
| 6535 | SLC6A8 | 575.16 | 657.24 | 493.09 | 0.75 | -0.41 | 6.02E-05 | 0.00581 |
| 117156 | SCGB3A2 | 22.14 | 14.49 | 29.78 | 2.05 | 1.04 | 6.36E-05 | 0.00610 |
| 79776 | ZFHX4 | 5.57 | 9.02 | 2.12 | 0.24 | -2.09 | 6.86E-05 | 0.00655 |
| 2180 | ACSL1 | 33718.68 | 28281.45 | 39155.91 | 1.38 | 0.47 | 6.91E-05 | 0.00657 |
| 7367 | UGT2B17 | 22.94 | 15.01 | 30.87 | 2.06 | 1.04 | 7.30E-05 | 0.00691 |
| 420 | ART4 | 4.01 | 1.55 | 6.47 | 4.17 | 2.06 | 7.77E-05 | 0.00732 |
| 8638 | OASL | 1000.64 | 853.05 | 1148.23 | 1.35 | 0.43 | 7.98E-05 | 0.00748 |
| 51733 | UPB1 | 187.98 | 156.02 | 219.94 | 1.41 | 0.50 | 8.33E-05 | 0.00776 |
| 9509 | ADAMTS2 | 22.70 | 31.78 | 13.62 | 0.43 | -1.22 | 8.36E-05 | 0.00776 |
| 366 | AQP9 | 25361.49 | 21542.95 | 29180.02 | 1.35 | 0.44 | 8.81E-05 | 0.00814 |
| 22846 | VASH1 | 1764.27 | 1993.00 | 1535.54 | 0.77 | -0.38 | 9.01E-05 | 0.00828 |
| 7380 | UPK3A | 97.87 | 115.18 | 80.56 | 0.70 | -0.52 | 9.13E-05 | 0.00835 |
| 2215 | FCGR3B | 81915.57 | 70230.05 | 93601.09 | 1.33 | 0.41 | 9.29E-05 | 0.00846 |
| 356 | FASLG | 435.47 | 502.51 | 368.43 | 0.73 | -0.45 | 9.34E-05 | 0.00846 |
| 266727 | MDGA1 | 510.28 | 447.43 | 573.12 | 1.28 | 0.36 | 0.00010 | 0.00908 |
| 1193 | CLIC2 | 279.11 | 234.24 | 323.98 | 1.38 | 0.47 | 0.00010 | 0.00916 |
| 8972 | MGAM | 12859.83 | 10910.61 | 14809.05 | 1.36 | 0.44 | 0.00010 | 0.00926 |
| 8876 | VNN1 | 1700.59 | 1435.76 | 1965.42 | 1.37 | 0.45 | 0.00010 | 0.00931 |
| 7053 | TGM3 | 181.28 | 144.91 | 217.65 | 1.50 | 0.59 | 0.00011 | 0.00967 |
| 84073 | MYCBPAP | 67.99 | 54.62 | 81.36 | 1.49 | 0.58 | 0.00011 | 0.00975 |
|  |  |  |  |  |  |  |  |  |
| Headers of the Table | |  |  |  |  |  |  |  |
| geneID | Gene Identification | |  |  |  |  |  |  |
| Gene Symbol | Official Symbol | |  |  |  |  |  |  |
| Base Mean | Mean normalized counts, averaged over all samples from both conditions | | | | | |  |  |
| Base Mean Best-Responders | Mean normalized counts from condition A | | | |  |  |  |  |
| Base Mean Worst-Responders | Mean normalized counts from condition B | | | |  |  |  |  |
| Fold Change | Fold change from condition A to B | | |  |  |  |  |  |
| Log2 Fold Change | The logarithm, to basis 2, of the fold change | | | |  |  |  |  |
| P value | P value for the statistical significance of this change | | | |  |  |  |  |
| Padj | P value adjusted for multiple testing with the Benjamini-Hochberg procedure, which controls false discovery rate | | | | | | | |
