# Supplementary material for: Blood Gene Expression Profile Predicts Response to Antipsychotics
Source: Front Mol Neurosci. 2018 Mar 6;11:73. doi: 10.3389/fnmol.2018.00073 (PMC5845714; doi:10.3389/fnmol.2018.00073)
Supplement: Supplementary file 3 [file Table_3.DOCX]

| **S3 Table. Differential expression between best-responders before and after medication** | | | | | | |  |  |
| --- | --- | --- | --- | --- | --- | --- | --- | --- |
| geneID | Gene Symbol | Base Mean | Base Mean Before Medication | Base Mean After Medication | Fold Change | Log2 Fold Change | Pval | Padj |
| 10562 | OLFM4 | 527.25 | 238.82 | 815.69 | 3.42 | 1.77 | 5.98E-57 | 1.16E-52 |
| 1088 | CEACAM8 | 644.61 | 328.23 | 960.99 | 2.93 | 1.55 | 3.87E-51 | 3.74E-47 |
| 4057 | LTF | 4321.76 | 2376.67 | 6266.85 | 2.64 | 1.40 | 2.14E-47 | 1.38E-43 |
| 10321 | CRISP3 | 310.05 | 162.33 | 457.76 | 2.82 | 1.50 | 9.85E-40 | 4.77E-36 |
| 154664 | ABCA13 | 314.93 | 162.86 | 467.00 | 2.87 | 1.52 | 3.51E-38 | 1.36E-34 |
| 3934 | LCN2 | 1192.72 | 719.07 | 1666.37 | 2.32 | 1.21 | 1.75E-37 | 5.64E-34 |
| 4317 | MMP8 | 536.88 | 309.44 | 764.32 | 2.47 | 1.30 | 4.75E-37 | 1.31E-33 |
| 9509 | ADAMTS2 | 82.00 | 129.42 | 34.58 | 0.27 | -1.90 | 1.06E-34 | 2.56E-31 |
| 100190986 | LOC100190986 | 37.37 | 7.98 | 66.77 | 8.37 | 3.07 | 1.83E-33 | 3.93E-30 |
| 1667 | DEFA1 | 2240.09 | 1550.00 | 2930.18 | 1.89 | 0.92 | 6.93E-33 | 1.12E-29 |
| 728358 | DEFA1B | 2240.09 | 1550.00 | 2930.18 | 1.89 | 0.92 | 6.93E-33 | 1.12E-29 |
| 1668 | DEFA3 | 2240.09 | 1550.00 | 2930.18 | 1.89 | 0.92 | 6.93E-33 | 1.12E-29 |
| 4680 | CEACAM6 | 465.65 | 283.11 | 648.19 | 2.29 | 1.20 | 1.75E-30 | 2.60E-27 |
| 100462981 | MTRNR2L2 | 80.75 | 26.81 | 134.69 | 5.02 | 2.33 | 5.77E-28 | 7.98E-25 |
| 1669 | DEFA4 | 460.27 | 305.47 | 615.08 | 2.01 | 1.01 | 5.90E-27 | 7.62E-24 |
| 100423062 | IGLL5 | 2936.15 | 4033.38 | 1838.92 | 0.46 | -1.13 | 4.32E-26 | 5.23E-23 |
| 671 | BPI | 943.03 | 635.54 | 1250.52 | 1.97 | 0.98 | 9.46E-25 | 1.08E-21 |
| 820 | CAMP | 681.78 | 451.33 | 912.23 | 2.02 | 1.02 | 5.41E-23 | 5.82E-20 |
| 4973 | OLR1 | 102.46 | 56.13 | 148.80 | 2.65 | 1.41 | 1.67E-22 | 1.70E-19 |
| 3512 | IGJ | 4839.59 | 6549.04 | 3130.14 | 0.48 | -1.07 | 4.25E-22 | 4.11E-19 |
| 221687 | RNF182 | 90.68 | 58.06 | 123.29 | 2.12 | 1.09 | 1.05E-20 | 9.71E-18 |
| 4353 | MPO | 869.31 | 624.26 | 1114.35 | 1.79 | 0.84 | 4.28E-18 | 3.76E-15 |
| 608 | TNFRSF17 | 166.13 | 232.16 | 100.09 | 0.43 | -1.21 | 3.03E-17 | 2.55E-14 |
| 566 | AZU1 | 280.90 | 197.36 | 364.44 | 1.85 | 0.88 | 4.62E-17 | 3.73E-14 |
| 81567 | TXNDC5 | 5718.95 | 7419.86 | 4018.04 | 0.54 | -0.88 | 3.43E-16 | 2.66E-13 |
| 1511 | CTSG | 136.40 | 91.82 | 180.97 | 1.97 | 0.98 | 1.41E-15 | 1.05E-12 |
| 3045 | HBD | 496.17 | 404.98 | 587.35 | 1.45 | 0.54 | 5.80E-15 | 4.16E-12 |
| 91543 | RSAD2 | 1559.22 | 2154.79 | 963.66 | 0.45 | -1.16 | 1.91E-14 | 1.32E-11 |
| 2038 | EPB42 | 438.40 | 351.83 | 524.98 | 1.49 | 0.58 | 6.93E-14 | 4.63E-11 |
| 51237 | MZB1 | 667.13 | 854.59 | 479.66 | 0.56 | -0.83 | 8.28E-14 | 5.34E-11 |
| 9172 | MYOM2 | 926.37 | 825.37 | 1027.38 | 1.24 | 0.32 | 1.55E-13 | 9.69E-11 |
| 27181 | SIGLEC8 | 555.24 | 437.31 | 673.17 | 1.54 | 0.62 | 1.85E-13 | 1.12E-10 |
| 212 | ALAS2 | 6731.39 | 5977.21 | 7485.58 | 1.25 | 0.32 | 2.42E-13 | 1.42E-10 |
| 1991 | ELANE | 227.68 | 168.49 | 286.87 | 1.70 | 0.77 | 2.69E-13 | 1.53E-10 |
| 6521 | SLC4A1 | 2780.57 | 2383.72 | 3177.42 | 1.33 | 0.41 | 3.41E-13 | 1.89E-10 |
| 10720 | UGT2B11 | 728.60 | 547.98 | 909.23 | 1.66 | 0.73 | 1.21E-12 | 6.48E-10 |
| 25893 | TRIM58 | 3988.88 | 3400.59 | 4577.17 | 1.35 | 0.43 | 2.42E-12 | 1.27E-09 |
| 5166 | PDK4 | 1850.32 | 2292.58 | 1408.06 | 0.61 | -0.70 | 3.83E-12 | 1.95E-09 |
| 932 | MS4A3 | 602.61 | 475.53 | 729.69 | 1.53 | 0.62 | 5.08E-12 | 2.52E-09 |
| 100526836 | BLOC1S5-TXNDC5 | 6727.06 | 8446.52 | 5007.60 | 0.59 | -0.75 | 5.24E-12 | 2.54E-09 |
| 8991 | SELENBP1 | 782.34 | 673.07 | 891.62 | 1.32 | 0.41 | 1.02E-11 | 4.81E-09 |
| 6948 | TCN2 | 398.72 | 501.98 | 295.46 | 0.59 | -0.76 | 1.18E-11 | 5.44E-09 |
| 3816 | KLK1 | 42.50 | 57.71 | 27.28 | 0.47 | -1.08 | 2.51E-11 | 1.13E-08 |
| 10900 | RUNDC3A | 491.48 | 407.32 | 575.64 | 1.41 | 0.50 | 2.78E-11 | 1.22E-08 |
| 2078 | ERG | 79.98 | 53.53 | 106.43 | 1.99 | 0.99 | 5.46E-11 | 2.30E-08 |
| 2731 | GLDC | 55.48 | 78.79 | 32.16 | 0.41 | -1.29 | 5.44E-11 | 2.30E-08 |
| 6947 | TCN1 | 325.82 | 249.03 | 402.61 | 1.62 | 0.69 | 5.81E-11 | 2.39E-08 |
| 712 | C1QA | 139.09 | 181.84 | 96.33 | 0.53 | -0.92 | 6.34E-11 | 2.56E-08 |
| 1308 | COL17A1 | 57.09 | 38.15 | 76.03 | 1.99 | 0.99 | 7.85E-11 | 3.10E-08 |
| 6037 | RNASE3 | 217.83 | 174.86 | 260.81 | 1.49 | 0.58 | 8.20E-11 | 3.18E-08 |
| 10410 | IFITM3 | 6051.52 | 7778.33 | 4324.71 | 0.56 | -0.85 | 1.01E-10 | 3.84E-08 |
| 246 | ALOX15 | 1512.48 | 1228.37 | 1796.59 | 1.46 | 0.55 | 1.46E-10 | 5.44E-08 |
| 3848 | KRT1 | 428.76 | 363.11 | 494.41 | 1.36 | 0.45 | 1.66E-10 | 6.06E-08 |
| 7145 | TNS1 | 1044.57 | 874.51 | 1214.62 | 1.39 | 0.47 | 2.18E-10 | 7.82E-08 |
| 5657 | PRTN3 | 85.11 | 57.66 | 112.56 | 1.95 | 0.97 | 6.19E-10 | 2.18E-07 |
| 7180 | CRISP2 | 7.97 | 2.01 | 13.93 | 6.93 | 2.79 | 1.03E-09 | 3.56E-07 |
| 26577 | PCOLCE2 | 17.97 | 8.44 | 27.51 | 3.26 | 1.71 | 1.60E-09 | 5.44E-07 |
| 4481 | MSR1 | 238.44 | 303.56 | 173.32 | 0.57 | -0.81 | 1.70E-09 | 5.67E-07 |
| 6622 | SNCA | 2356.50 | 2095.76 | 2617.25 | 1.25 | 0.32 | 2.43E-09 | 7.96E-07 |
| 6768 | ST14 | 1223.30 | 1482.70 | 963.90 | 0.65 | -0.62 | 5.07E-09 | 1.64E-06 |
| 1118 | CHIT1 | 135.25 | 96.32 | 174.19 | 1.81 | 0.85 | 5.89E-09 | 1.87E-06 |
| 644248 | RP11-67M1.1 | 369.37 | 319.64 | 419.10 | 1.31 | 0.39 | 6.31E-09 | 1.97E-06 |
| 146439 | CCDC64B | 208.36 | 239.74 | 176.97 | 0.74 | -0.44 | 9.11E-09 | 2.80E-06 |
| 3437 | IFIT3 | 5089.19 | 6312.60 | 3865.77 | 0.61 | -0.71 | 1.34E-08 | 4.05E-06 |
| 10215 | OLIG2 | 170.58 | 138.99 | 202.17 | 1.45 | 0.54 | 2.41E-08 | 7.18E-06 |
| 710 | SERPING1 | 673.67 | 839.51 | 507.84 | 0.60 | -0.73 | 2.48E-08 | 7.28E-06 |
| 9672 | SDC3 | 231.67 | 283.87 | 179.47 | 0.63 | -0.66 | 2.74E-08 | 7.92E-06 |
| 713 | C1QB | 80.71 | 106.26 | 55.15 | 0.52 | -0.95 | 3.88E-08 | 1.10E-05 |
| 116071 | BATF2 | 269.94 | 335.40 | 204.48 | 0.61 | -0.71 | 4.46E-08 | 1.25E-05 |
| 55512 | SMPD3 | 2046.17 | 1734.81 | 2357.54 | 1.36 | 0.44 | 5.26E-08 | 1.46E-05 |
| 765 | CA6 | 707.78 | 572.49 | 843.07 | 1.47 | 0.56 | 7.06E-08 | 1.92E-05 |
| 1958 | EGR1 | 163.74 | 204.30 | 123.18 | 0.60 | -0.73 | 7.48E-08 | 2.01E-05 |
| 3620 | IDO1 | 393.56 | 343.93 | 443.19 | 1.29 | 0.37 | 7.78E-08 | 2.06E-05 |
| 2039 | DMTN | 4657.11 | 4072.02 | 5242.20 | 1.29 | 0.36 | 1.03E-07 | 2.70E-05 |
| 1278 | COL1A2 | 6.95 | 12.37 | 1.53 | 0.12 | -3.01 | 1.05E-07 | 2.72E-05 |
| 3568 | IL5RA | 944.93 | 820.37 | 1069.49 | 1.30 | 0.38 | 1.09E-07 | 2.78E-05 |
| 3434 | IFIT1 | 2511.13 | 3198.41 | 1823.84 | 0.57 | -0.81 | 1.32E-07 | 3.33E-05 |
| 94031 | HTRA3 | 30.32 | 18.60 | 42.03 | 2.26 | 1.18 | 1.60E-07 | 3.96E-05 |
| 343171 | OR2W3 | 282.87 | 248.51 | 317.24 | 1.28 | 0.35 | 1.70E-07 | 4.17E-05 |
| 8685 | MARCO | 520.18 | 634.25 | 406.11 | 0.64 | -0.64 | 2.21E-07 | 5.35E-05 |
| 54674 | LRRN3 | 1318.62 | 1147.83 | 1489.41 | 1.30 | 0.38 | 2.48E-07 | 5.92E-05 |
| 23495 | TNFRSF13B | 181.06 | 215.47 | 146.64 | 0.68 | -0.56 | 2.71E-07 | 6.41E-05 |
| 200132 | TCTEX1D1 | 34.36 | 24.18 | 44.53 | 1.84 | 0.88 | 5.23E-07 | 0.00012 |
| 116285 | ACSM1 | 231.12 | 189.41 | 272.82 | 1.44 | 0.53 | 5.19E-07 | 0.00012 |
| 64105 | CENPK | 726.66 | 624.71 | 828.61 | 1.33 | 0.41 | 5.22E-07 | 0.00012 |
| 6382 | SDC1 | 19.92 | 29.73 | 10.11 | 0.34 | -1.56 | 5.52E-07 | 0.00012 |
| 51208 | CLDN18 | 25.34 | 15.77 | 34.91 | 2.21 | 1.15 | 5.89E-07 | 0.00013 |
| 1374 | CPT1A | 4130.94 | 4783.06 | 3478.82 | 0.73 | -0.46 | 6.40E-07 | 0.00014 |
| 6279 | S100A8 | 46177.58 | 54173.12 | 38182.04 | 0.70 | -0.50 | 8.11E-07 | 0.00018 |
| 714 | C1QC | 29.76 | 41.10 | 18.42 | 0.45 | -1.16 | 8.57E-07 | 0.00018 |
| 139189 | DGKK | 147.05 | 115.17 | 178.93 | 1.55 | 0.64 | 8.93E-07 | 0.00019 |
| 3624 | INHBA | 9.64 | 4.09 | 15.19 | 3.71 | 1.89 | 9.11E-07 | 0.00019 |
| 439996 | IFIT1B | 231.41 | 207.57 | 255.26 | 1.23 | 0.30 | 1.07E-06 | 0.00022 |
| 57156 | TMEM63C | 315.33 | 337.98 | 292.68 | 0.87 | -0.21 | 1.07E-06 | 0.00022 |
| 54094 | C21orf15 | 273.94 | 222.83 | 325.04 | 1.46 | 0.54 | 1.15E-06 | 0.00023 |
| 26790 | SNORD58B | 456.03 | 548.92 | 363.15 | 0.66 | -0.60 | 1.19E-06 | 0.00024 |
| 9636 | ISG15 | 1315.98 | 1608.45 | 1023.51 | 0.64 | -0.65 | 1.26E-06 | 0.00025 |
| 1053 | CEBPE | 330.22 | 284.10 | 376.33 | 1.32 | 0.41 | 1.34E-06 | 0.00026 |
| 717 | C2 | 305.74 | 367.37 | 244.11 | 0.66 | -0.59 | 1.42E-06 | 0.00028 |
| 26509 | MYOF | 1564.55 | 1854.17 | 1274.94 | 0.69 | -0.54 | 1.47E-06 | 0.00028 |
| 401399 | PRRT4 | 65.54 | 47.16 | 83.91 | 1.78 | 0.83 | 1.68E-06 | 0.00032 |
| 11326 | VSIG4 | 183.58 | 217.95 | 149.21 | 0.68 | -0.55 | 1.68E-06 | 0.00032 |
| 51348 | KLRF1 | 2790.49 | 2292.59 | 3288.39 | 1.43 | 0.52 | 1.83E-06 | 0.00034 |
| 759 | CA1 | 370.80 | 335.82 | 405.78 | 1.21 | 0.27 | 1.82E-06 | 0.00034 |
| 1232 | CCR3 | 2605.55 | 2323.03 | 2888.07 | 1.24 | 0.31 | 1.94E-06 | 0.00036 |
| 100874264 | AOAH-IT1 | 33.76 | 46.10 | 21.43 | 0.46 | -1.11 | 2.12E-06 | 0.00039 |
| 283120 | H19 | 35.74 | 23.61 | 47.87 | 2.03 | 1.02 | 2.57E-06 | 0.00046 |
| 10437 | IFI30 | 20962.76 | 24541.67 | 17383.85 | 0.71 | -0.50 | 2.68E-06 | 0.00048 |
| 5273 | SERPINB10 | 66.59 | 50.46 | 82.73 | 1.64 | 0.71 | 3.05E-06 | 0.00054 |
| 645843 | TMEM14E | 11.06 | 17.53 | 4.59 | 0.26 | -1.93 | 3.09E-06 | 0.00054 |
| 10581 | IFITM2 | 39106.55 | 43532.98 | 34680.12 | 0.80 | -0.33 | 3.78E-06 | 0.00066 |
| 598 | BCL2L1 | 7030.78 | 6357.02 | 7704.53 | 1.21 | 0.28 | 3.93E-06 | 0.00068 |
| 5909 | RAP1GAP | 67.22 | 53.77 | 80.67 | 1.50 | 0.59 | 4.02E-06 | 0.00069 |
| 28 | ABO | 135.40 | 109.62 | 161.18 | 1.47 | 0.56 | 4.09E-06 | 0.00069 |
| 83943 | IMMP2L | 1668.83 | 1473.07 | 1864.59 | 1.27 | 0.34 | 4.28E-06 | 0.00072 |
| 3047 | HBG1 | 214.99 | 255.83 | 174.14 | 0.68 | -0.55 | 4.34E-06 | 0.00072 |
| 260429 | PRSS33 | 776.40 | 681.83 | 870.97 | 1.28 | 0.35 | 4.38E-06 | 0.00073 |
| 2209 | FCGR1A | 791.68 | 928.64 | 654.72 | 0.71 | -0.50 | 4.46E-06 | 0.00073 |
| 441864 | TARM1 | 19.66 | 11.26 | 28.05 | 2.49 | 1.32 | 5.95E-06 | 0.00097 |
| 23251 | KIAA1024 | 139.02 | 115.22 | 162.82 | 1.41 | 0.50 | 6.07E-06 | 0.00098 |
| 991 | CDC20 | 65.24 | 83.26 | 47.22 | 0.57 | -0.82 | 6.23E-06 | 0.00100 |
| 4883 | NPR3 | 41.67 | 31.92 | 51.43 | 1.61 | 0.69 | 6.35E-06 | 0.00101 |
| 125875 | CLDND2 | 288.46 | 229.98 | 346.94 | 1.51 | 0.59 | 6.82E-06 | 0.00107 |
| 79365 | BHLHE41 | 148.37 | 174.65 | 122.08 | 0.70 | -0.52 | 7.04E-06 | 0.00110 |
| 54490 | UGT2B28 | 56.10 | 42.46 | 69.75 | 1.64 | 0.72 | 7.75E-06 | 0.00120 |
| 149345 | SHISA4 | 139.74 | 111.55 | 167.93 | 1.51 | 0.59 | 8.14E-06 | 0.00125 |
| 8347 | HIST1H2BC | 237.36 | 268.00 | 206.72 | 0.77 | -0.37 | 8.63E-06 | 0.00132 |
| 59340 | HRH4 | 380.84 | 331.20 | 430.47 | 1.30 | 0.38 | 8.86E-06 | 0.00134 |
| 6280 | S100A9 | 115029.06 | 132140.14 | 97917.98 | 0.74 | -0.43 | 8.97E-06 | 0.00135 |
| 26807 | SNORD43 | 593.62 | 671.75 | 515.49 | 0.77 | -0.38 | 1.06E-05 | 0.00157 |
| 6038 | RNASE4 | 439.21 | 509.41 | 369.00 | 0.72 | -0.47 | 1.09E-05 | 0.00160 |
| 51339 | DACT1 | 160.51 | 135.30 | 185.71 | 1.37 | 0.46 | 1.19E-05 | 0.00173 |
| 3162 | HMOX1 | 3001.45 | 3442.32 | 2560.58 | 0.74 | -0.43 | 1.18E-05 | 0.00173 |
| 5097 | PCDH1 | 135.32 | 110.73 | 159.92 | 1.44 | 0.53 | 1.26E-05 | 0.00181 |
| 201305 | SPNS3 | 1487.71 | 1320.47 | 1654.95 | 1.25 | 0.33 | 1.34E-05 | 0.00191 |
| 9935 | MAFB | 2327.45 | 2718.66 | 1936.23 | 0.71 | -0.49 | 1.35E-05 | 0.00193 |
| 59352 | LGR6 | 512.95 | 423.46 | 602.45 | 1.42 | 0.51 | 1.38E-05 | 0.00195 |
| 199675 | C19orf59 | 767.81 | 885.48 | 650.14 | 0.73 | -0.45 | 1.39E-05 | 0.00195 |
| 83999 | KREMEN1 | 1572.53 | 1767.55 | 1377.50 | 0.78 | -0.36 | 1.50E-05 | 0.00209 |
| 6614 | SIGLEC1 | 1029.94 | 1291.11 | 768.76 | 0.60 | -0.75 | 1.61E-05 | 0.00222 |
| 1281 | COL3A1 | 4.94 | 8.84 | 1.04 | 0.12 | -3.09 | 1.99E-05 | 0.00274 |
| 2030 | SLC29A1 | 1174.62 | 1050.48 | 1298.76 | 1.24 | 0.31 | 2.01E-05 | 0.00274 |
| 440603 | BCL2L15 | 103.77 | 82.24 | 125.31 | 1.52 | 0.61 | 2.20E-05 | 0.00298 |
| 83742 | MARVELD1 | 794.82 | 917.57 | 672.08 | 0.73 | -0.45 | 2.45E-05 | 0.00329 |
| 2322 | FLT3 | 639.28 | 752.72 | 525.84 | 0.70 | -0.52 | 2.63E-05 | 0.00350 |
| 2900 | GRIK4 | 68.22 | 53.13 | 83.30 | 1.57 | 0.65 | 2.69E-05 | 0.00356 |
| 1277 | COL1A1 | 10.78 | 16.65 | 4.92 | 0.30 | -1.76 | 2.83E-05 | 0.00373 |
| 360226 | PRSS41 | 115.04 | 95.29 | 134.79 | 1.41 | 0.50 | 2.97E-05 | 0.00388 |
| 59283 | CACNG8 | 256.93 | 226.10 | 287.77 | 1.27 | 0.35 | 3.37E-05 | 0.00438 |
| 432 | ASGR1 | 451.71 | 518.17 | 385.24 | 0.74 | -0.43 | 3.66E-05 | 0.00472 |
| 51629 | SLC25A39 | 13521.96 | 12353.97 | 14689.94 | 1.19 | 0.25 | 3.92E-05 | 0.00503 |
| 151473 | SLC16A14 | 69.00 | 56.80 | 81.21 | 1.43 | 0.52 | 4.19E-05 | 0.00533 |
| 440689 | HIST2H2BF | 1639.72 | 1839.23 | 1440.21 | 0.78 | -0.35 | 4.31E-05 | 0.00545 |
| 401258 | RAB44 | 1152.66 | 1009.02 | 1296.31 | 1.28 | 0.36 | 4.35E-05 | 0.00547 |
| 4938 | OAS1 | 3580.04 | 4236.24 | 2923.84 | 0.69 | -0.53 | 5.61E-05 | 0.00700 |
| 83869 | TTTY14 | 1712.41 | 1493.33 | 1931.49 | 1.29 | 0.37 | 5.64E-05 | 0.00700 |
| 7225 | TRPC6 | 75.00 | 60.46 | 89.53 | 1.48 | 0.57 | 5.83E-05 | 0.00718 |
| 29065 | ASAP1-IT1 | 18.80 | 25.86 | 11.74 | 0.45 | -1.14 | 5.88E-05 | 0.00721 |
| 10107 | TRIM10 | 84.73 | 71.37 | 98.10 | 1.37 | 0.46 | 6.02E-05 | 0.00733 |
| 57801 | HES4 | 78.76 | 68.17 | 89.34 | 1.31 | 0.39 | 6.12E-05 | 0.00741 |
| 719 | C3AR1 | 1858.34 | 2144.70 | 1571.99 | 0.73 | -0.45 | 6.20E-05 | 0.00746 |
| 114990 | VASN | 113.09 | 89.10 | 137.08 | 1.54 | 0.62 | 6.27E-05 | 0.00749 |
| 83483 | PLVAP | 181.81 | 159.71 | 203.91 | 1.28 | 0.35 | 6.41E-05 | 0.00761 |
| 6322 | SCML1 | 804.05 | 705.00 | 903.09 | 1.28 | 0.36 | 6.51E-05 | 0.00769 |
| 59286 | UBL5 | 1723.99 | 1958.38 | 1489.60 | 0.76 | -0.39 | 6.66E-05 | 0.00781 |
| 10501 | SEMA6B | 60.75 | 45.36 | 76.13 | 1.68 | 0.75 | 6.84E-05 | 0.00798 |
| 2359 | FPR3 | 218.52 | 266.64 | 170.40 | 0.64 | -0.65 | 7.01E-05 | 0.00808 |
| 100133941 | CD24 | 1685.69 | 1471.54 | 1899.84 | 1.29 | 0.37 | 6.98E-05 | 0.00808 |
| 146857 | SLFN13 | 1866.76 | 1610.53 | 2122.98 | 1.32 | 0.40 | 7.46E-05 | 0.00854 |
| 10417 | SPON2 | 4919.37 | 4142.82 | 5695.93 | 1.37 | 0.46 | 7.85E-05 | 0.00893 |
| 91319 | DERL3 | 456.48 | 533.16 | 379.80 | 0.71 | -0.49 | 7.97E-05 | 0.00903 |
| 10409 | BASP1 | 8417.78 | 9266.47 | 7569.09 | 0.82 | -0.29 | 8.13E-05 | 0.00915 |
| 22807 | IKZF2 | 1715.23 | 1524.67 | 1905.78 | 1.25 | 0.32 | 8.24E-05 | 0.00922 |
| 6241 | RRM2 | 252.66 | 293.84 | 211.48 | 0.72 | -0.47 | 8.47E-05 | 0.00942 |
| 51314 | NME8 | 727.95 | 613.30 | 842.60 | 1.37 | 0.46 | 8.98E-05 | 0.00993 |
| 8510 | MMP23B | 74.83 | 54.69 | 94.97 | 1.74 | 0.80 | 9.03E-05 | 0.00994 |
|  |  |  |  |  |  |  |  |  |
| Headers of the Table | |  |  |  |  |  |  |  |
| geneID | Gene Identification | |  |  |  |  |  |  |
| Gene Symbol | Official Symbol | |  |  |  |  |  |  |
| Base Mean | Mean normalized counts, averaged over all samples from both conditions | | | | | |  |  |
| Base Mean Before Medication | Mean normalized counts from condition A | | | |  |  |  |  |
| Base Mean After Medication | Mean normalized counts from condition B | | | |  |  |  |  |
| Fold Change | Fold change from condition A to B | | |  |  |  |  |  |
| Log2 Fold Change | The logarithm, to basis 2, of the fold change | | | |  |  |  |  |
| P value | P value for the statistical significance of this change | | | |  |  |  |  |
| Padj | P value adjusted for multiple testing with the Benjamini-Hochberg procedure, which controls false discovery rate | | | | | | | |
