# Supplementary material for: Blood Gene Expression Profile Predicts Response to Antipsychotics
Source: Front Mol Neurosci. 2018 Mar 6;11:73. doi: 10.3389/fnmol.2018.00073 (PMC5845714; doi:10.3389/fnmol.2018.00073)
Supplement: Supplementary file 4 [file Table_4.DOCX]

| **S4 Table. Differential expression between worst-responders before and after medication** | | | | | | | |  |
| --- | --- | --- | --- | --- | --- | --- | --- | --- |
| geneID | Gene Symbol | Base Mean | Base Mean Before Medication | Base Mean After Medication | Fold Change | Log2 Fold Change | Pval | Padj |
| 100462981 | MTRNR2L2 | 502.53 | 24.13 | 980.92 | 40.65 | 5.35 | 5.59E-240 | 1.08E-235 |
| 9381 | OTOF | 140.04 | 218.40 | 61.69 | 0.28 | -1.82 | 1.68E-52 | 1.62E-48 |
| 3429 | IFI27 | 77.61 | 116.33 | 38.90 | 0.33 | -1.58 | 1.10E-37 | 7.10E-34 |
| 100190986 | LOC100190986 | 35.88 | 9.86 | 61.91 | 6.28 | 2.65 | 5.76E-30 | 2.78E-26 |
| 1719 | DHFR | 1128.99 | 651.12 | 1606.86 | 2.47 | 1.30 | 5.58E-23 | 2.16E-19 |
| 9509 | ADAMTS2 | 34.40 | 55.22 | 13.58 | 0.25 | -2.02 | 6.72E-20 | 2.16E-16 |
| 29065 | ASAP1-IT1 | 15.84 | 27.43 | 4.26 | 0.16 | -2.69 | 6.38E-19 | 1.76E-15 |
| 100463486 | MTRNR2L8 | 158.50 | 88.80 | 228.19 | 2.57 | 1.36 | 8.79E-18 | 2.12E-14 |
| 5139 | PDE3A | 856.13 | 1108.75 | 603.51 | 0.54 | -0.88 | 7.22E-15 | 1.55E-11 |
| 554226 | ANKRD30BL | 320.68 | 421.95 | 219.41 | 0.52 | -0.94 | 3.69E-14 | 7.12E-11 |
| 3488 | IGFBP5 | 8.60 | 15.10 | 2.10 | 0.14 | -2.84 | 1.47E-12 | 2.59E-09 |
| 81796 | SLCO5A1 | 19740.04 | 24475.09 | 15004.99 | 0.61 | -0.71 | 2.52E-10 | 4.06E-07 |
| 212 | ALAS2 | 4829.24 | 6329.93 | 3328.56 | 0.53 | -0.93 | 3.70E-10 | 5.49E-07 |
| 164045 | HFM1 | 34344.31 | 42282.92 | 26405.69 | 0.62 | -0.68 | 8.20E-10 | 1.13E-06 |
| 23500 | DAAM2 | 346.92 | 407.58 | 286.26 | 0.70 | -0.51 | 7.24E-09 | 9.32E-06 |
| 124912 | SPACA3 | 14.80 | 21.58 | 8.02 | 0.37 | -1.43 | 7.95E-09 | 9.60E-06 |
| 6614 | SIGLEC1 | 1596.30 | 1727.71 | 1464.89 | 0.85 | -0.24 | 6.85E-08 | 7.78E-05 |
| 246 | ALOX15 | 1071.40 | 898.95 | 1243.84 | 1.38 | 0.47 | 2.85E-07 | 0.00031 |
| 1740 | DLG2 | 5040.87 | 6052.13 | 4029.61 | 0.67 | -0.59 | 3.83E-07 | 0.00039 |
| 221687 | RNF182 | 59.99 | 82.40 | 37.58 | 0.46 | -1.13 | 9.83E-07 | 0.00095 |
| 3620 | IDO1 | 260.27 | 209.83 | 310.70 | 1.48 | 0.57 | 1.25E-06 | 0.00115 |
| 3047 | HBG1 | 284.19 | 366.59 | 201.79 | 0.55 | -0.86 | 1.91E-06 | 0.00167 |
| 714 | C1QC | 31.75 | 40.26 | 23.25 | 0.58 | -0.79 | 2.38E-06 | 0.00200 |
|  |  |  |  |  |  |  |  |  |
| Headers of the Table | |  |  |  |  |  |  |  |
| geneID | Gene Identification | |  |  |  |  |  |  |
| Gene Symbol | Official Symbol | |  |  |  |  |  |  |
| Base Mean | Mean normalized counts, averaged over all samples from both conditions | | | | | |  |  |
| Base Mean Before Medication | Mean normalized counts from condition A | | | |  |  |  |  |
| Base Mean After Medication | Mean normalized counts from condition B | | | |  |  |  |  |
| Fold Change | Fold change from condition A to B | | |  |  |  |  |  |
| Log2 Fold Change | The logarithm, to basis 2, of the fold change | | | |  |  |  |  |
| P value | P value for the statistical significance of this change | | | |  |  |  |  |
| Padj | P value adjusted for multiple testing with the Benjamini-Hochberg procedure, which controls false discovery rate | | | | | | | |
