# Supplementary material for: Blood Gene Expression Profile Predicts Response to Antipsychotics
Source: Front Mol Neurosci. 2018 Mar 6;11:73. doi: 10.3389/fnmol.2018.00073 (PMC5845714; doi:10.3389/fnmol.2018.00073)
Supplement: Supplementary file 5 [file Table_5.DOCX]

| **S5 Table. The 30 genes with the highest predictive power** | | | | |
| --- | --- | --- | --- | --- |
| Gene symbol | Chr | Start | End | Gini |
| SLC9A3 | chr5 | 473333 | 524549 | 0.90692249 |
| HMOX1 | chr22 | 35777059 | 35790207 | 0.895315136 |
| SLC22A16 | chr6 | 110745891 | 110797844 | 0.505405963 |
| LOC284581 | chr1 | 205831206 | 205865215 | 0.501917214 |
| PF4V1 | chr4 | 74719012 | 74720198 | 0.392855982 |
| GSTT1 | chr22 | 24376138 | 24384284 | 0.380503482 |
| DLC1 | chr8 | 12940871 | 13372429 | 0.327980015 |
| AQP10 | chr1 | 154293591 | 154297801 | 0.303985142 |
| NLRP2 | chr19 | 55476651 | 55512510 | 0.287145296 |
| C17orf97 | chr17 | 260117 | 264457 | 0.260801575 |
| CRIP2 | chr14 | 105939274 | 105946507 | 0.258219847 |
| EGFL7 | chr9 | 139553307 | 139567130 | 0.23700338 |
| LOC642846 | chr12 | 9436252 | 9466684 | 0.235587821 |
| ATOH8 | chr2 | 85980908 | 86018506 | 0.231554415 |
| FMN1 | chr15 | 33057744 | 33486934 | 0.229543432 |
| BTNL3 | chr5 | 180415844 | 180433727 | 0.228373919 |
| DGKK | chrX | 50108405 | 50213737 | 0.210864272 |
| FAM153B | chr5 | 175490711 | 175541801 | 0.199406607 |
| C4BPA | chr1 | 207277606 | 207318317 | 0.195035924 |
| TRIM6 | chr11 | 5617330 | 5634188 | 0.193156146 |
| KRT72 | chr12 | 52979372 | 52995322 | 0.189711245 |
| IL8 | chr4 | 74606222 | 74609433 | 0.18594549 |
| PTGDS | chr9 | 139871955 | 139876194 | 0.184917648 |
| NTNG2 | chr9 | 135037333 | 135118220 | 0.182725824 |
| TMEM63C | chr14 | 77648101 | 77725838 | 0.176103025 |
| LIPN | chr10 | 90521162 | 90537999 | 0.170802146 |
| VWF | chr12 | 6058039 | 6233836 | 0.168301614 |
| ANK2 | chr4 | 113739238 | 114304896 | 0.157175476 |
| CENPK | chr5 | 64813592 | 64858995 | 0.154019124 |
| SIGLEC1 | chr20 | 3667616 | 3687775 | 0.15289179 |
|  |  |  |  |  |
| Headers of the Table |  |  |  |  |
| Gene symbol | Official Symbol | |  |  |
| Chr | Chromosome | |  |  |
| Start | Gene Start Coordinate | | |  |
| End | Gene End Coordinate | |  |  |
| Gini | Gini variable importance measures reflect the mean decrease in impurity by splits of a given variable in the classification tree, weighted by the proportion of samples reaching that node | | | |
